# Cytosporone B as a Biological Preservative: Purification, Fungicidal Activity and Mechanism of Action against *Geotrichum citri-aurantii*

**DOI:** 10.3390/biom9040125

**Published:** 2019-03-29

**Authors:** Chunxiao Yin, Hongxin Liu, Yang Shan, Vijai Kumar Gupta, Yueming Jiang, Weimin Zhang, Haibo Tan, Liang Gong

**Affiliations:** 1 Key Laboratory of Plant Resource Conservation and Sustainable Utilization, Guangdong Provincial Key Laboratory of Applied Botany, South China Botanical Garden, Chinese Academy of Sciences, Guangzhou 510650, China; chunxiaoyin@scbg.ac.cn (C.Y.); ymjiang@scbg.ac.cn (Y.J.); tanhaibo@scbg.ac.cn (H.T.); 2 Long Ping Branch, Graduate School of Hunan University, Changsha 410125, China; sy6302@sohu.com; 3 Guangdong Open Laboratory of Applied Microbiology, Guangdong Provincial Key Laboratory of Microbial Culture Collection and Application, State Key Laboratory of Applied Microbiology Southern China, Guangdong Institute of Microbiology, Guangzhou 510070, China; liuhx@gdim.cn; 4 Department of Chemistry and Biotechnology, ERA Chair of Green Chemistry, Tallinn University of Technology, 12618 Tallinn, Estonia; 5 Key Laboratory of Post-Harvest Handling of Fruits, Ministry of Agriculture, Guangzhou 510650, China

**Keywords:** cytosporone B, citrus decay, *Geotrichum citri-aurantii*, RNA-Seq, mode of action

## Abstract

To prevent citrus decay caused by *Geotrichum citri-aurantii*, 12 natural products were isolated from two endophytic fungi, in which cytosporone B was shown to have excellent bioactivity for control of *G. citri-aurantii* with median effect concentration (EC_50_) of 26.11 μg/mL and minimum inhibitory concentration (MIC) of 105 μg/mL, and also significantly reduced the decay of sugar orange during the in vivo trials. In addition, cytosporone B could alter the morphology of *G. citri-aurantii* by causing distortion of the mycelia and loss of membrane integrity. Differentially expressed genes (DEGs) between cytosporone B-treated and -untreated samples were revealed by Illumina sequencing, including 3540 unigenes. Gene Ontology (GO) and Kyoto Encyclopedia of Genes and Genomes (KEGG) analyses showed that most DEGs were related to metabolic production and cell membrane. These findings suggest cytosporone B is a promising biological preservative to control citrus decay and reveal the action mechanism of cytosporone B in relation to the destruction of the fungal cell membrane at both morphological and molecular levels.

## 1. Introduction

Citrus is one of the most important fruit crops with a global production of 146.4 million tons in 2016 [1]. Each year, over 25% of produced citrus fruits are lost by postharvest decay, much of which is caused by fungal infections [2]. Fungal infection may occur in a wound of citrus due to poor handling, packaging or storage conditions.

Citrus sour rot is one of the most serious citrus diseases caused by a heterothallic fungus *Geotrichum citri-aurantii* with the teleomorph of *Galactomyces citri-aurantii* [3]. Iminoctadine tris (albesilate) is the leading commercially applied fungicide for control of *G. citri-aurantii* in China, but long-term use of a single synthetic fungicide may induce pathogen resistance against many site-specific fungicides [4]. It is also well-recognized that the use of chemical and synthetic fungicides to control plant pathogens has many disadvantages and limitations, especially with respect to environmental and residual issues. Hence, previous studies have focused on the application of natural products for the control of *G. citri-aurantii*, demonstrating that essential oils and/or plant organic extracts can be effective in preventing postharvest citrus sour rot diseases [5,6] and that yeasts secreting hydrolytic enzymes have great potential for control of *G. citri-aurantii* [7]. Indeed, microbial, plant and animal-derived compounds have been proposed as potential alternatives to synthetic fungicides for reducing the decay of postharvest citrus [8,9].

Cytosporone B is a fungal polyketide chemical that was first isolated from an endophytic fungus *Cytospora* sp. and was shown to have a wide range of antitumor and antimicrobic activities [10]. Cytosporone B was previously reported as an effective SPI-1 (Serine Protease Inhibitor-1) inhibitor that may have potential in drug development against antibiotic-resistant *Salmonella* [11]. Recently, cytosporone B was used as a specific agonist of NR4A1 (Nuclear Receptor Subfamily 4 Group A member 1) to successfully treat mice infected with the influenza virus, suggesting its capacity for regulating inflammatory and immune response [12]. Furthermore, cytosporone B may serve as a novel antifibrotic agent for fibrosis in the vocal folds [13]. Even though cytosporone B has been demonstrated to exhibit diverse biological activities, its mode of action remains to be investigated. It was only reported that the molecular mechanism of cytosporone B is associated with inflammatory disease. For example, Zhan et al. (2008) showed that cytosporone B physically binds to nuclear orphan receptor Nur77 resulting in the stimulation of its transcriptional activity and increased expression of NR4A1 [14] and that treatment with cytosporone B for mice infected with IAV (Influenza A virus) reduced the lung viral loads and significantly improved pulmonary function, in part due to the stimulation of type 1 IFN (Interferon) synthesis in the presence of AMs (Alveolas macrophage) [12]. However, the mechanism of the action of cytosporone B against plant pathogens has never been reported.

Recently, RNA-Seq technology has been developed to reveal the presence and amount of RNA in an organism during development and/or under different conditions. The development of RNA-Seq technology has launched a novel approach for investigating the action mechanisms of antifungal compounds. For example, the action mechanism of 2-phenylethanol against *Penicillium italicum*, a pathogen of citrus blue mold, has been studied using RNA-Seq, showing that a number of essential pathways associated with the cell cycle and cell death participate in inhibiting *P. italicum* [15]. RNA-seq was applied to determine the transcriptomic response of *Cronobacter sakazakii* to garlic-driven organosulfur compounds, ajoene and diallyl sulfide under the sublethal concentrations [16]. To the best of our knowledge, the present work is the first report on the expression profile of the genes in *G. citri-aurantii* under the cytosporone B treatment.

In the present study, four octaketides, three aromatic compounds, two monoterpenes, and three polyketides were isolated from two endophytes *Phomopsis phyllanthicola* A658 and *Cytospora rhizophorae* A761. All of these compounds were evaluated against *G. citri-aurantii* in vitro. Furthermore, the most active compound (cytosporone B) was further investigated for the protective effect on orange fruit suffering from *G. citri-aurantii* infection. In addition, RNA-seq was used to systematically study the genotypic difference of *G. citri-aurantii* under the cytosporone B treatment. This study demonstrated an effective phytopathogenic fungal inhibitor, cytosporone B that may have a novel mode of action.

## 2. Materials and Methods

### 2.1. General Experiment Procedures

The NMR spectra were acquired using a Bruker Avance 500 MHz MHz NMR spectrometer with TMS as an internal standard (Bruker, Fallanden, Switzerland). ESIMS data were collected on an Agilent Technologies 1290-6430A Triple Quad LC/MS (Agilent Technologies, Palo Alto, CA, USA). Preparative HPLC separations were carried out using a YMC-pack ODS-A column (250 × 20 mm, 5 µm, and 12 nm, YMC Co., Ltd., Kyoto, Japan). Semi-preparative HPLC separations were performed utilizing a YMC-pack ODS-A/AQ column (250 × 10 mm, 5 µm, and 12 nm, YMC Co., Ltd., Kyoto, Japan) and a YMC-pack Cellulose-SB column (250 × 10 mm, 5 µm, and 12 nm, YMC Co., Ltd., Kyoto, Japan). Column chromatography were performed with silica gel (200–300 mesh, Qingdao Marine Chemical Inc., Qingdao, China) and Sephadex LH-20 (Amersham Biosciences, Uppsala, Sweden), respectively. Thin-layer chromatography (TLC) was conducted with precoated glass plates GF-254 (Merck KGaA, Darmastadt, Germany).

### 2.2. Fungi and Culture

*G. citri-aurantii* was isolated from rotten citrus fruit and kindly provided by Pro. Hu MY (South China Agricultural University, Guangzhou, China). The fungus was maintained in a potato dextrose agar (PDA) culture medium at 28 °C. The endophytic fungal strains *Phomopsis phyllanthicola* A658 and *Cytospora rhizophorae* A761 were isolated from the tissues of *Pogostemon cablin* and *Morinda officinalis*, respectively, which are widely cultivated in Guangdong province and are well known as “Guang Huo Xiang” and “Ba Ji Tian” in traditional Chinese medicine. The plant tissues were cut to 0.5 × 0.5 cm^2^ segments. The segments were surface-sterilized by immersing them in 75% ethanol for 20 s followed by treatment with 0.01% mercury solution for 60 s. Then, the segments were washed in sterile water (60 s) and plated on potato dextrose agar (PDA) medium amended with 40 mg/L ampicillin and 40 mg/L kanamycin. Petri dishes were incubated in a biochemical incubator at 28 °C and observed for 10 days.

The strain A761 was isolated from the leaves of *Morinda officinalis*, which was collected from Gaoyao city of Guangdong province in January 2015. The strain was identified by sequence analysis of rRNA ITS (internal transcribed spacer) region. The sequence of ITS region of the fungus A761 has been submitted to GenBank (Accession No. KU529867). By using BLAST (nucleotide sequence comparison program) to search the GenBank database, A761 was found to have 99.5% similarity with *Cytospora rhizophorae* M225 (Accession No. KR056292). The strain A658 was isolated from the stems of *Pogostemon cablin*, which was collected from Yangchun city of Guangdong province, China, in October 2012. The strain was identified by sequence analysis of rRNA ITS region. The sequence of ITS region of the fungal strain A658 has been submitted to GenBank (Accession No. KF498871). By using BLAST (nucleotide sequence comparison program) to search the GenBank database, A658 was found to have 99.80% similarity with *Phomopsis phyllanthicola* A6 (Accession No. EF488373). The strain A658 and A761 were preserved at the Guangdong Provincial Key Laboratory of Microbial Culture Collection and Application, Guangdong Institute of Microbiology.

### 2.3. Fermentation, Extraction, and Isolation

*P. phyllanthicola* A658 and *C. rhizophorae* A761 were cultured in a potato dextrose broth (PDB, potato 20%, glucose 2%, K_2_HPO_4_ 0.3%, MgSO_4_•7H_2_O 0.15%, vitamin B 10 mg/L). The A658 and A761 fungi were maintained in a potato dextrose agar (PDA) medium at 28 °C for 5 days, and then three pieces (0.5 × 0.5 cm^2^) of mycelial agar plugs were inoculated into 20 × 500 mL Erlenmeyer flasks, each containing 250 mL of PDB. After 4 days of incubation at 28 °C on a rotary shaker at 120 rpm, 25 mL samples of A761 and A658 were aseptically transferred into a total of 100 flasks (1000 mL capacity) each containing 500 mL of PDB. The subsequent liquid cultivation was performed for 7 days at 28 °C and 120 rpm on a rotary shaker.

Each culture (50 L) of A658 and A761 were centrifuged to provide the broth (supernatant) and mycelia (precipitate), respectively. The broth was exhaustively extracted with EtOAc four times, and then, the ethanolic extracts were combined and evaporated under reduced pressure at a temperature not exceeding 40 °C to yield the dark brown gum (20 g) of A658 and (26 g) of A761.

The crude EtOAc extract of A658 was subjected to silica gel column chromatography (*n*-hexane/EtOAc, 1:0→0:1, *v*/*v*) to afford five fractions (Fr.1–Fr.5). Fr. 2 was subjected to CC on Sephadex LH-20 (CH_2_Cl_2_/MeOH, 1:1, *v*/*v*) to yield three subfractions Fr.2-1 to Fr. 2-3. Fr.2-1 was purified by silica gel flash column chromatography (*n*-hexane/EtOAc, 5:1→1:1, *v*/*v*) and semiprep-HPLC (MeOH/H_2_O, 67:33, *v*/*v*, 3 mL/min) to give compounds **6** (2.0 mg, *t*_R_ = 8.0 min) and **10** (4.0 mg, *t*_R_ = 10.5 min). Fr. 2-2 was further purified by silica gel flash column chromatography (*n*-hexane/EtOAc, 5:1→2:1, *v*/*v*) to yield compound **1** (120.0 mg) and Fr 2-2-1. Fr. 2-3 was further purified by silica gel flash column chromatography (*n*-hexane/EtOAc, 5:1→1:1, *v*/*v*) to yield compound **5** (5.0 mg) and Fr.2-3-2. Fr.2-2-1 was further purified by silica gel flash column chromatography (*n*-hexane/EtOAc, 5:1→1:1, *v*/*v*) to yield Fr. 2-2-1-1. Fr. 2-2-1-1 was further purified by semiprep-HPLC (MeOH/H_2_O, 45:54, *v*/*v*, 3 mL/min) to give three subfractions, Fr.2-2-1-1-1 to Fr.2-2-1-1-3. Fr.2-2-1-1-1 was further purified by semiprep-HPLC (MeOH/H_2_O, 60:40, *v*/*v*, 3 mL/min) to afford compounds **8** (20 mg, *t*_R_ = 11.0 min) and Fr.2-2-1-1-1-1. Fr.2-2-1-1-1-1 was further purified by semiprep-HPLC (AcOH/H_2_O, 34:66, *v*/*v*, 3 mL/min) to give **7** (5.0 mg, *t*_R_ = 8.3 min) and **9** (5.0 mg, *t*_R_ = 11.8 min). Fr.2-3-2 was further purified by semiprep-HPLC (MeOH/H_2_O, 46:54, *v*/*v*, 3 mL/min) to afford compounds **11** (2.0 mg, *t*_R_ = 15.3 min) and **12** (20 mg, *t*_R_ = 17.0 min).

The crude EtOAc extract of A761 was subjected to reversed-phase silica gel C_18_ (MeOH/H_2_O, 30%→100%) column chromatography to afford 6 fractions (Fr.1–Fr.6). Fr. 5 was further subjected to CC on Sephadex LH-20 (MeOH) and was followed by silica gel column chromatography and semiprep-HPLC (ACN/H_2_O, 50:50, *v*/*v*, 3 mL/min) to obtain compounds **2** (4.0 mg, *t*_R_ = 7.2 min), **3** (10 mg, *t*_R_ = 8.3 min), and **4** (6.0 mg, *t*_R_ = 9.0 min).

### 2.4. Bioassays

#### 2.4.1. In Vitro Assays

The in vitro assays were conducted with two-step tests. First, each isolated compound was tested at the concentration of 50 µg/mL to determine the compound that showed the highest effectiveness for restraining the mycelial growth of *G. citri-aurantii* according to our previous report [17]. Second, the fungicidal activity of cytosporone B, which was the most efficient compound, was further determined by inhibiting the radial growth of fungi on PDA in the presence of a series of concentrations (2, 4, 8, 16, 32, and 64 µg/mL). The fungicide prochloraz was used as a positive control, while the negative control containing 0.1% (*v*/*v*) DMSO was utilized in this experiment. The inhibition ratio (%) of colony growth was recorded as follows: [(average diameter of control − average diameter of treatment)/average diameter of control] × 100. The experiment was performed in triplicate. Minimum inhibitory concentration (MIC) was tested by microscopic observation of mycelial growth in 96-well microtiter plates as reported by Karim et al. (2017) [18].

#### 2.4.2. In Vivo Assays

The in vivo bioactivity of cytosporone B against *G. citri-aurantii* was analyzed for a sugar orange. The citrus fruits without physical injuries and visual infections were chosen for the in vivo assays. Prior to the experimental use, the fruits were wiped by cotton with 75% ethanol and then air-dried. By using a sterile needle, each fruit was wounded (5 mm deep and 2 mm wide) at four positions in both sides of the equator. Then, the fruits were dipped for 5 min in the cytosporone B solution (250 µg/mL and 500 µg/mL), prochloraz solution (250 µg/mL and 500 µg/mL) and sterile distilled water (negative control). Then, 10 µL of a spore suspension (1 × 10^6^ conidia/mL) was pipetted into each wound, and the fruits were placed into an incubator to maintain stationary temperature (25 °C) and relative humidity (~95%) for 5 days. Each treatment was replicated three times with 20 fruits per replication. The decay rate was calculated as follows:

Decay rate (%) = [(number of rotten wounds/number of total wounds)] × 100.

### 2.5. Scanning Electron Microscopy

The surface hyphal morphology of *G. citri-aurantii* was observed by scanning electron microscopy (SEM) according to our previous reports [14].

### 2.6. Determination of Cytoplasmic Membrane Integrity

A 0.5 mL (10^7^ spores/mL) conidial suspension of *G. citri-aurantii* was incubated in potato dextrose (PD) liquid medium (1.5 mL) containing different concentrations of cytosporone B (0, 25, 50, and 100 µg/mL) at 28 °C for 2.5 h. The spores were collected by centrifugation at 8000 g for 5 min at room temperature and were stained with 10 µg/mL propidium iodide (PI; Sigma-Aldrich) for 15 min at 37 °C. After removing the supernatant by centrifugation, and washing twice with phosphate-buffer saline (PBS), the concentration of the conidial suspensions was determined with a hemacytometer and adjusted to 10^6^ spores/mL with PBS. The spores were observed, and the images were collected using a Leica TCS SP8 X (Leica, Solms, Germany) white light laser confocal microscope.

### 2.7. Transcriptional Analysis

#### 2.7.1. RNA Extraction and Illumina Sequencing

*G. citri-aurantii* was cultured in a liquid PDA medium with a median lethal dose of cytosporone B (26.11 µg/mL) for 5 days, and then the mycelia were collected for total RNA extraction using HiPure Fungal RNA Mini Kit (Magen, Guangzhou, China). NanoDrop 2000 (Thermo Scientific, Wilmington, DE, USA), Qubit 2.0 (Carlsbad, CA, USA) and Aglient 2100 (Agilent Technologies, CA, UAS) were used to evaluate the quantity, quality, and integrity of the total RNAs. The qualified RNA was frozen in liquid nitrogen immediately and then stored at −80 °C. Three biological replicates for the treatment and control were collected, respectively.

For RNA-seq, the RNAs were enriched by magnetic beads with Oligo (dT) and then mixed with the fragmentation buffer to prepare their short fragments. cDNA library was synthesized using the mRNA fragments and random hexamer primers. For quality control, Agilent 2100 Bioanaylzer and ABI StepOnePlus Real-Time PCR System were used for the quantitative and qualitative analysis of the sample library. The libraries were sequenced by HiSeqX-ten (Illumina, Santiago, CA, USA), with a read length of 150 bp. Clean reads were obtained by removing the reads containing adaptors or unknown nucleotides larger than 5%. The raw data have been stored in the NCBI Sequence Read Archive database with the accession number of PRJNA487514.

#### 2.7.2. Sequence Assembly, Annotation, and Expression Analysis

Due to the lack of the reference genome for *G. citri-aurantii*, the de novo transcriptome was combined and assembled by using the clean data from all of the above samples according to the Trinity methods [19]. The filtered unigenes were estimated based on the percentage of mapped reads in each library, read length distribution, and saturation analysis of the mapped reads. Then, the unigenes were annotated with BLAST against the database of NR (NCBI nonredundant protein database, http://www.ncbi.nlm.nih.gov/), GO (Gene Ontology, http://www.geneontology.org/), COG (Clusters of Orthologous Groups, http://clovr.org/docs/clusters-of-orthologous-groups-cogs/), and KEGG (Kyoto Encyclopedia of Genes and Genomes, http://www.genome.jp/tools/kaas/) databases. RPKM (reads per kilobase of exon model per million mapped reads) was utilized to quantify the gene expression level [20]. Pearson’s correlation coefficient (PCC) was calculated to measure the linear correlation among the samples [21]. DESeq was adopted to analyze the differential expression of genes (DEGs) [22]. A gene with the *P*-value of 0.05 and log2 (fold change) of 2 was considered to be significantly differently expressed between the two conditions.

### 2.8. Quantitative Real-Time PCR Analysis

Total RNA was extracted from cytosporone B treated and untreated samples as described above. The total RNA was treated with DNaseI (Takara, Shiga, Japan) and then subjected to reverse transcript to cDNA using a reverse transcription system (Takara, Shiga, Japan). The quantitative real-time PCR (qRT-PCR) was performed using an Applied Biosystems 7500 Real-time PCR system (Applied Biosystems) with SYBR Premix Ex Taq (Takara, Shiga, Japan). Each reaction contained 20 ng of the first-strand cDNA as the template, in a total reaction mixture volume of 20 mL. The following conditions were used for amplification: 95 °C for 20 s, 40 cycles of PCR amplification at 95 °C for 10 s, 60 °C for 30 s, and 70 °C for 1 s. Gene-specific primers, shown in Supplementary file 1, were used for detecting the relative quantification of each gene, and β-Actin was used as an internal control for normalization. The qRT-PCR expression levels were compared based on the mean of three independent experimental replicates. Calculation of the relative expression level was performed using the 2^–ΔΔCT^ method [23].

### 2.9. Statistical Analysis

Graphpad software 5.0 (La Jolla, CA USA) was used to perform statistical analysis using Tukey’s test with the *p*-values ≤ 0.05 considered as statistically significant.

## 3. Results

### 3.1. Structure Identification of Compounds ***1***–***12***

**Figure 1:**
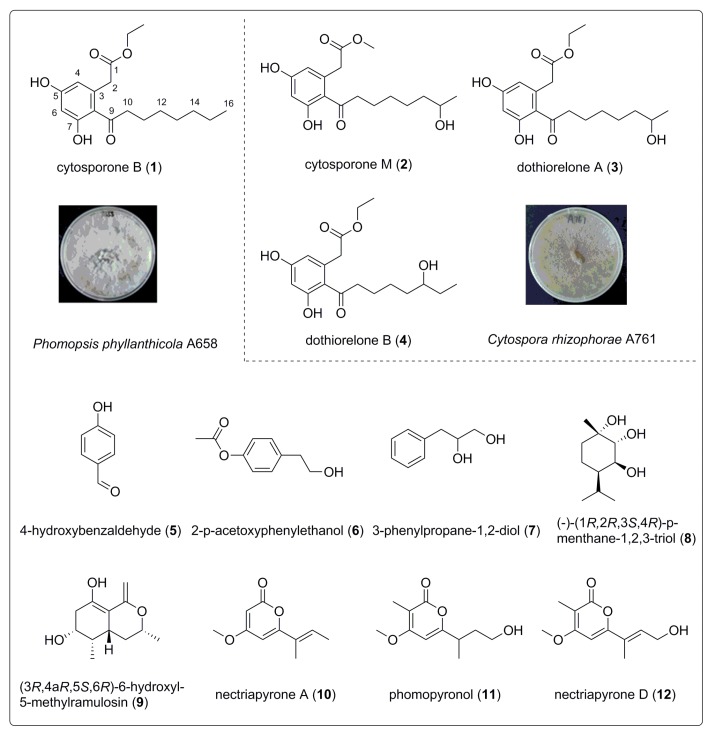
Structures of compounds **1**–**12** (compounds **1** and **5**–**12** were isolated from A658, and compounds **2**–**4** were isolated from A761).

The phytochemical study on the EtOAc extract of the fungi *Phomopsis phyllanthicola* A658 and *Cytospora rhizophorae* A761 resulted in the isolation of the twelve known compounds (Figure 1), including four octaketides—cytosporone B (**1**) [10], cytosporone M (**2**) [24], dothiorelone A (**3**), and dothiorelone B (**4**) [25]; three aromatic compounds—4-hydroxybenzaldehyde (**5**) [26], 2-p-acetoxyphenylethanol (**6**) [27], and 3-phenylpropane-1,2-diol (**7**) [28]; two monoterpenes,—(–)-(1*R*,2*R*,3*S*,4*R*)-p-menthane-1,2,3-triol (**8**) [29] and (3*R*,4a*R*,5*S*,6*R*)-6-hydroxyl-5-methylramulosin (**9**) [30]; and three polyketides—nectriapyrone A (**10**) [31], phomopyronol (**11**) [28], and nectriapyrone D (**12**) [32].

Spectroscopic data for four octaketides:

Cytosporone B (**1**): pale yellow oil; ^1^H NMR (500 MHz, CDCl_3_): *δ* 6.27 (d, *J* = 2 Hz, H-4, 6), 4.21 (2H, q, *J* = 7.0 Hz, H-17), 3.81 (2H, s, H-2), 2.84 (2H, t, *J* = 7.0 Hz, H-10), 1.69 (m, H-11), 1.30 (8H, m, H-12-H-15), 1.30 (3H, t, *J* = 7.0 Hz, H-18), 0.89 (3H, t, *J* = 7.0 Hz, H-16); ^13^C NMR (125 MHz, CDCl_3_): 171.9 (C-1), 40.6 (C-2), 136.4 (C-3), 116.9 (C-4), 160.6 (C-5), 103.2 (C-6), 163.7 (C-7), 112.7 (C-8), 206.9 (C-9), 43.5 (C-10), 25.0 (C-11), 29.2 (C-12), 29.1 (C-13), 31.7 (C-14), 22.6 (C-15), 14.1 (C-16), 61.7 (C-17), 14.0 (C-18).

Cytosporone M (**2**): colorless oil; ^1^H NMR (500 MHz, CD_3_OD): *δ* 6.28 (d, *J* = 2.3 Hz, H-4), 6.27 (d, *J* = 2.3 Hz, H-6), 3.72 (m, H-15), 3.60 (2H, s, H-2), 2.93 (2H, t, *J* = 7 Hz, H-10), 1.65 (2H, m, H-11), 1.35-1.40 (6H, m, H-12, 13, 14), 1.16 (3H, d, *J* = 6.2 Hz, H-16); ^13^C NMR (125 MHz, CD_3_OD): 172.6 (C-1), 43.8 (C-2), 135.6 (C-3), 119.8 (C-4), 158.4 (C-5), 101.4 (C-6), 160.0 (C-7), 110.4 (C-8), 207.6 (C-9), 43.8 (C-10), 39.0 (C-11), 31.7 (C-12), 25.3 (C-13), 38.7 (C-14), 67.2 (C-15), 22.1 (C-16), 50.9 (OMe).

Dothiorelone A (**3**): amorphous powder; ^1^H NMR (500 MHz, CD_3_OD): *δ* 6.28 (d, *J* = 2.3 Hz, H-4), 6.22 (d, *J* = 2.3 Hz, H-6), 4.13 (q, *J* = 7.1 Hz, H-17), 3.72 (m, H-15), 3.60 (2H, s, H-2), 2.93 (2H, t, *J* = 7.1 Hz, H-10), 1.64 (2H, m, H-11), 1.26-1.33 (6H, m, H-12, 13, 14), 1.26 (3H, t, *J* = 7.1 Hz, H-18), 1.16 (3H, d, *J* = 6.2 Hz, H-16); ^13^C NMR (125 MHz, CD_3_OD): 173.5 (C-1), 40.5 (C-2), 135.6 (C-3), 111.7 (C-4), 161.3 (C-5), 102.3 (C-6), 159.8 (C-7), 121.7 (C-8), 208.9 (C-9), 45.2 (C-10), 25.5 (C-11), 30.5 (C-12), 26.7 (C-13), 40.0 (C-14), 68.5 (C-15), 23.5 (C-16), 61.8 (C-17), 14.5 (C-18).

Dothiorelone B (**4**): colorless oil; ^1^H NMR (500 MHz, CD_3_OD): *δ* 6.28 (d, *J* = 2.3 Hz, H-4), 6.21 (d, *J* = 2.3 Hz, H-6), 4.13 (2H, q, *J* = 7.1 Hz, H-17), 3.60 (2H, s, H-2), 3.46 (m, H-14), 2.95 (2H, t, *J* = 7.1 Hz, H-10), 1.65 (2H, m, H-11), 1.39-1.50 (6H, m, H-12, 13, 14), 1.26 (3H, t, *J* = 7.1 Hz, H-18), 0.95 (3H, d, *J* = 5.3 Hz, H-16); ^13^C NMR (125 MHz, CD_3_OD): 172.2 (C-1), 41.6 (C-2), 135.6 (C-3), 110.4 (C-4), 159.9 (C-5), 101.4 (C-6), 158.4 (C-7), 110. (C-8), 207.5 (C-9), 43.8 (C-10), 25.2 (C-11), 29.2 (C-12), 36.4 (C-13), 72.4 (C-14), 29.7 (C-15), 9.9 (C-16), 60.5 (C-17), 29.6 (C-18).

The chemical structures of all of the compounds are shown in Figure 1, and additional NMR (Nuclear Magnetic Resonance) spectra data are provided in Figures S1–S25.

### 3.2. In Vivo and In Vitro Activity of Cytosporone B

**Figure 2:**
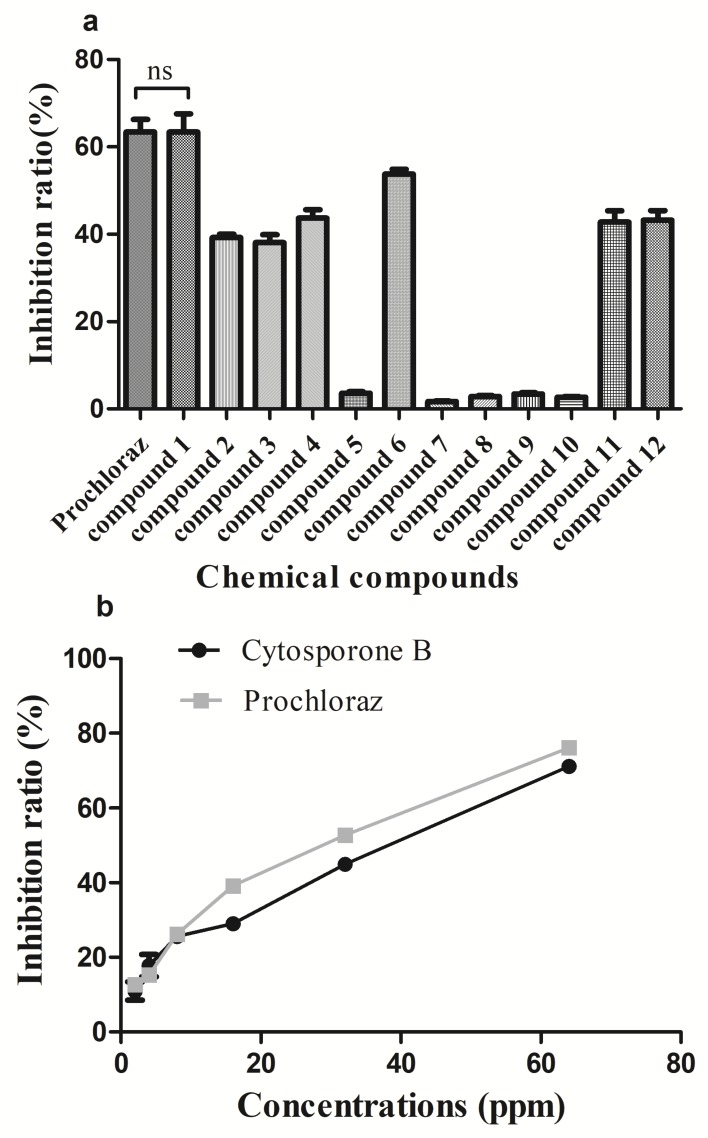
Effect of isolated chemicals (**a**) and cytosporone B (**b**) on mycelial growth of *Geotrichum citri-aurantii* on potato dextrose agar plates. *Vertical bars* represent the standard error of the means; ns means no significant difference.

**Figure 3:**
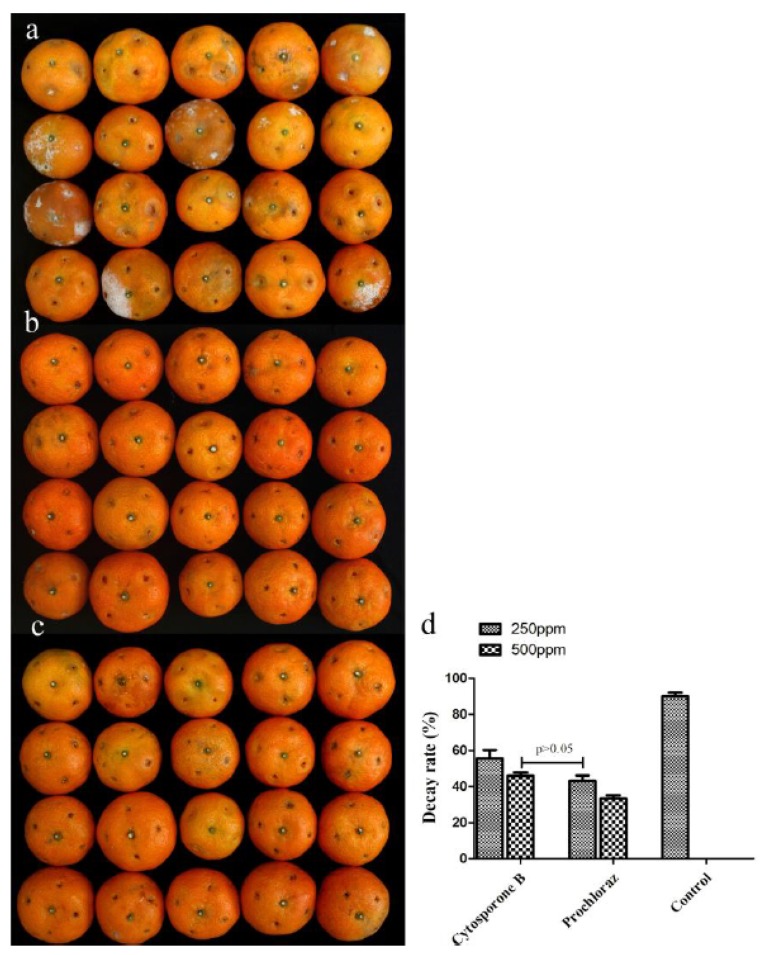
Influence of cytosporone B treatment on the development of *Geotrichum citri-aurantii* in sugar orange. (**a**) Negative control (0.1% DMSO), (**b**) cytosporone B at 500 µg/mL(ppm), (**c**) prochloraz at 500 µg/mL (ppm) and (**d**) percentage of the control effects. *Vertical bars* represent standard error of the means; *p* > 0.05 means no significant difference.

As shown in Figure 2a, some of the isolated compounds showed high growth inhibition to *G. citri-aurantii*, among which the inhibition ratio of cytosporone B (compound 1) reached 63.4% which was similar to that of the positive control. The inhibition efficiency of cytosporone B against *G. citri-aurantii* showed a dose-dependent behavior with the EC_50_ = 26.11 µg/mL, while the commercialized fungicide prochloraz has EC_50_ =18.92 µg/mL (Figure 2b). Additionally, the MICs were evaluated as 105 µg/mL and 95 µg/mL for cytosporone B and prochloraz, respectively. Moreover, cytosporone B showed a promising protection effect on sugar orange inoculated with *G. citri-aurantii*, suggesting that the in vivo control efficiency of cytosporone B at the concentration of 500 µg/mL was comparable to that of prochloraz at the concentration of 250 µg/mL (Figure 3).

### 3.3. Membrane Integrity of G. citri-aurantii under the Treatment of Cytosporone B

**Figure 4:**
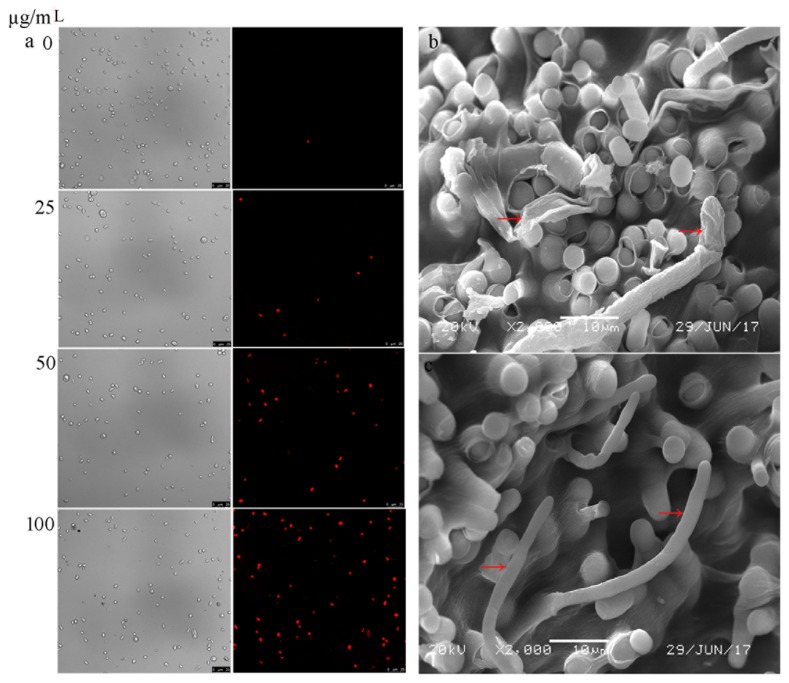
Plasma membranes of the spores that were damaged by cytosporone B represented by red fluorescence (**a**); scanning electron microscopy of hypha and spores of *G. citri-aurantii* underlying the treatment of cytosporone B (**b**); and negative control (**c**).

To investigate the mechanism of cytosporone B induced inhibition for *G. citri-aurantii*, propidium iodide (PI) was used to determine the membrane integrity of *G. citri-aurantii* upon exposure to cytosporone B. PI is a fluorescent dye that cannot penetrate an intact plasma membrane, and, therefore, can only enter a damaged plasma membrane and show red fluorescent under UV excitation. As shown in Figure 4a, few spores were stained by PI in the control sample, while the number of PI stained spores increased following the cytosporone B treatment, suggesting a close correlation between the fraction of the spores that lost the membrane integrity and the cytosporone B dosage. Furthermore, SEM images showed the morphological structural damages suffered by *G. citri-aurantii* after the cytosporone B treatment (Figure 4b). These results suggested that cytosporone B may inhibit mycelial growth of *G. citri-aurantii* by causing cell wall and plasma membrane disturbance, and finally, cytosporone B led to the spore death of *G. citri-aurantii*.

### 3.4. Transcriptome Sequencing and DGEs Analysis of G. citri-aurantii

**Figure 5:**
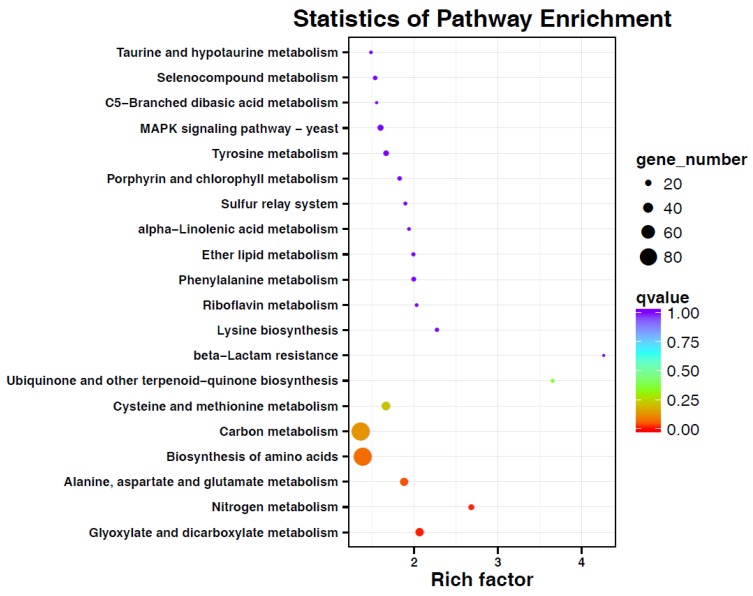
Kyoto Encyclopedia of Genes and Genomes (KEGG) enrichment analyses of differentially expressed genes in *G. citri-aurantii* underlying the treatment of cytosporone B.

Illumina sequencing was carried out by sequencing by the synthesis principle, resulting in the generation of 150,414,078 clean reads with the Q30 of more than 86.94% (Table S1). After assembling, 55,906 unigenes were obtained with the N50 of 1130 (Table S2). Among these, 37,302 unigenes were annotated by the above-mentioned BLAST (Supplementary file 3, Table S3). The statistical analysis showed that the two samples from cytosporone B-treated or -untreated *G. citri-aurantii* showed good correlation (R^2^ >0.8); hence, these were utilized for the subsequent DGEs analysis. A total of 3540 DGEs were found between the two groups, in which 1412 unigenes were significantly upregulated and 2128 unigenes were downregulated (Figure S1), suggesting cytosporone B was biased in favor of inhibiting the gene expression. Among the DEGs, the top three COG function classifications were amino acid transport and metabolism (158); carbohydrate transport and metabolism (134); and translation, ribosomal structure, and biogenesis (123), except that of the general function prediction only (Figure S2). The top three KOG function classifications were posttranslational modification, protein turnover, chaperones (192); signal transduction mechanisms (145), and amino acid transport and metabolism (124), except that of general function prediction only (Supplementary file 3, Figure S3). The GO description showed that the most significant terms were the nutrient reservoir activity, growth, and extracellular matrix part (3, Figure S4). All the annotated genes were mapped to the terms in the KEGG database to find the significantly enriched genes associated with the metabolic or signal transduction pathways. Thirty-one DEGs in terms of nitrogen metabolism, 17 DEGs in terms of glyoxylate and dicarboxylate metabolism, and 6 DEGs in terms of ubiquinone and other terpenoid-quinone biosynthesis were identified as predominant enrichment processes in the comparison of cytosporone B treated versus untreated *G. citri-aurantii* (Figure 5).

### 3.5. Identification of Genes Related to Amino Acid Synthesis and Metabolism

**Figure 6:**
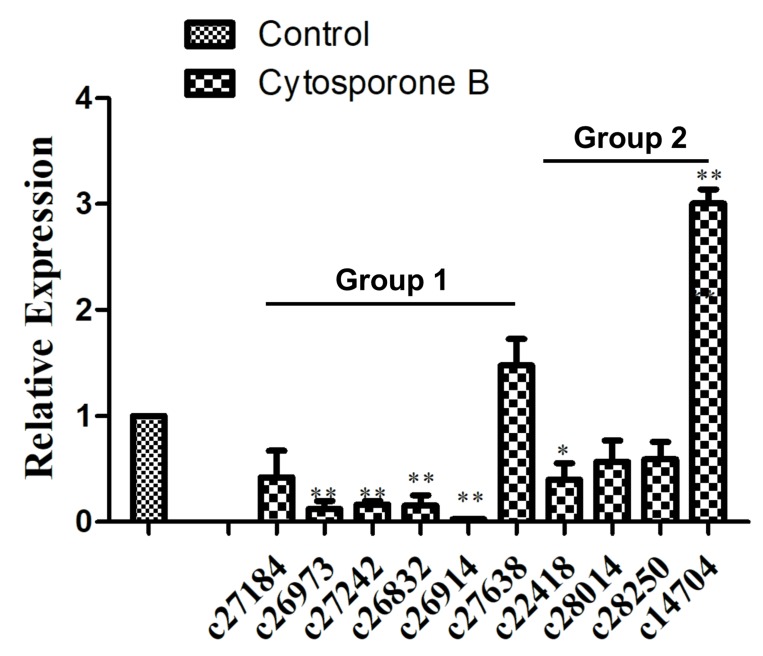
Relative expression levels of selected target genes of cytosporone B in *G. citri-aurantii*. Gene expression in control group was set as 1. Group 1 means the genes involved in amino acid synthesis and metabolism, and group 2 means the genes involved in signal transduction mechanisms. Each datum point represents a mean ± standard deviation (n = 3), and the values marked by the asterisks are significantly different (* *p* < 0.05; ** *p* < 0.01).

Based on the above gene functional annotation, it was shown that genes associated with amino acid synthesis and metabolism might be closely related to *G. citri-aurantii* under the treatments. As shown in supplementary file 4, 128 unigenes were annotated to amino acid transport and metabolism, of which 109 and 19 unigenes were significantly inhibited and induced, respectively, by cytosporone B. In addition, 10 of these genes were randomly chosen for qPCR analysis, and six of them were successfully amplified in the experiments. As shown in Figure 6, c27184 annotated as aromatic amino acid aminotransferase I was downregulated 2.38-fold, c26973 annotated as glutamine synthethase was downregulated 8.18-fold and c27242 annotated as glutamate dehydrogenase was downregulated 6.09-fold, and only c27638 annotated as histidinol dehydrogenase was upregulated 1.47-fold, which was an opposite result to that obtained for RNA-Seq.

### 3.6. Identification of Genes Related to Signal Transduction Mechanisms

Signal transduction may also play essential roles in cytosporone B induced growth inhibition of *G. citri-aurantii*. As shown in Supplementary file 5, 174 unigenes were annotated to the signal transduction mechanisms, of which 163 and 11 unigenes were significantly inhibited and induced by cytosporone B, respectively. Ten of these genes were randomly chosen, and all successfully amplified in qPCR experiments. For example, the expressions of c22418, c28014, and c28250 were inhibited in the treated samples, which was confirmed by qPCR in agreement with the RNA-Seq results (Figure 6). In the case of the MAPK signaling pathway, six of seven unigenes were downregulated, and only c14704—annotated as phosphatidylinositol signaling—was upregulated 3.0-fold in the cytosporone B treated samples compared to the control samples (Figure 6).

## 4. Discussion

The emergence of microorganism resistance is the main reason for the continuous development of new fungicides. Prochloraz, an imidazole fungicide, is widely used to control the growth of fungi in China. Hence, it was used as a positive control in this study; however, the extensive use of prochloraz has given rise to the significant development of drug-resistant strains. Hellin et al. (2018) reported that a tebuconazole-adapted *Fusarium culmorum* strain had developed cross-resistance to all demethylation inhibitors including prochloraz [33]. *P. digitatum*, a severe postharvest disease of citrus fruit, has developed high resistant to prochloraz in Hubei Province, China [34]. Therefore, it is still necessary to discover new chemicals as an alternative for the management of plant pathogens. *Phomopsis* is an important phytopathogenic genus that contains more than 900 species according to the wide range of hosts [35]. In the present study, to search for active antifungal lead compounds as efficient fungicides for reducing decay of postharvest citrus, the chemical constitution study on the culture broth of endophytes *P. phyllanthicola* A658 and *C. rhizophorae* A761 led to the isolation of 12 natural products with four octaketides, three aromatic compounds, two monoterpenes, and three polyketides. The biological evaluation toward the inhibition of *G. citri-aurantii* clarified that most of these isolated natural compounds showed potent antifungal activity at the concentration of 50 µg/mL. In particular, cytosporone B exhibited a significant growth inhibition with the potency similar to that of the commercialized fungicide prochloraz (Figure 2 and Figure 3). Moreover, the yield of compound **1** was 120 mg/50 L, indicating that such compound could be scaled for production purposes.

The results from the antifungal screening toward *G. citri-aurantii* also demonstrated a preliminary structure–activity relationship, wherein octaketides were confirmed to be the predominating components responsive for the potent antifungal activity of the endophytes *P. phyllanthicola* A658 and *C. rhizophorae* A761. Moreover, the hydroxyl functionality in the acyl chain of cytosporone B derivatives was observed to play a critical role in their antifungal activity, and its existence would tend to decrease the antifungal potency of cytosporone B dramatically. However, the location of the hydroxyl group and the simple replacement of the ethyl moiety by the methyl moiety in the ester group for the cytosporone B derivative appeared to have little influence on the antifungal potency (2-4). These results imply an inverse relationship between the strength of the fungal activity and the existence of the hydroxyl functionality in the acyl tails on the cytosporone B derivatives.

To investigate the mechanism of cytosporone B-induced growth inhibition of *G. citri-aurantii*, the integrity of the plasma membrane of *G. citri-aurantii* after the cytosporone B treatment was evaluated. Previous studies indicated that chemicals against *Botrytis cinerea* could destroy the integrity of the plasma membrane, such as boron [36] and cinnamic acid [37]. Our results showed that the effect was positively correlated with the number of spores that lost membrane integrity and the concentrations of cytosporone B, suggesting that an increasing amount of spores were killed with the increased cytosporone B dosage. In addition, SEM images of *G. citri-aurantii* exposed to cytosporone B showed marked morphological changes including hyphal cell membrane collapsing and cell lysis. It was reported that some biological or abiotic substances with antimicrobial activity could cause similar symptoms, such as *Lactobacillus harbinensis* against *Yarrowia lipolytica* [38] and stilbene derivatives against phytopathogenic fungi [39]. These results suggested for the first time that cytosporone B showed antimicrobial activity against *G. citri-aurantii* by disrupting the cell membrane integrity and causing the leakage of cell components.

To develop novel antifungal agents, it is essential to understand the mechanism of their actions at a molecular level. To date, it has been reported that cytosporone B can be an effective SPI-1 inhibitor in antibiotic-resistant *Salmonella* [11] and stimulate Nur77-dependent transactivational activity to inhibit cancer cell growth [40]. However, there is no molecular evidence regarding its mode of action for fungi; our results suggest that cytosporone B could affect the expression of a very large number of genes in *G. citri-aurantii* and that these genes were significantly clustered to metabolic production and cell membrane (Figure 5 and Figures S2–S4), especial in the category of amino acid transport and metabolism and signal transduction mechanisms. We infer that cytosporone B may change the production of secondary metabolites in *G. citri-aurantii*, because transport and metabolism of amino acids play several critical roles for providing organic nitrogen to the biosynthesis of essential metabolites in organisms. For example, aromatic amino acid aminotransferase I (c27184) that was significantly inhibited in the cytosporone B treated samples has been demonstrated to be related to 2-phenylethanol [41] and tyrosol biosynthesis [42]. The downregulation of glutamine synthethase (c26973) may account for the decreased production of bioactive secondary metabolites in *G. citri-aurantii*, because it was reported that glutamine synthethase plays an important role in the production of fusaristatin A in *Fusarium graminearum* [43] and for spinosad production in *Saccharopolyspora spinosa* [44]. A total of 109 unigenes associated with amino acid transport and metabolism were significantly downregulated in the cytosporone B treated samples, especially some unigenes c26669, c25801, and c24950 associated with the integral component of membrane were completely inhibited in the samples (supplementary file 4), suggesting cytosporone B may severely disorder protein metabolism and integrity of cell membrane in *G. citri-aurantii*, and this should be one of the reasons for the cytosporone B caused death of *G. citri-aurantii*. However, further investigation of the exact mode of action is needed.

Targeting a signal transduction protein was considered as a novel strategy for developing potential drugs for medically important fungi [45]. For an example, histidine kinase (HK) is a transmembrane protein that plays a role in signal transduction and that can be phosphorylated by ATP upon receiving intra and extracellular signals in bacteria, archaea, fungi, and plants; but HK is not found in humans. Hence, it could be an important target for drug discovery in human pathogenic microorganism [46]. Here, four putative HK has significantly downregulated the expression in the cytosporone B treated samples, suggesting that cytosporone B may be an inhibitor of HK. Indeed, this capability of cytosporone B can be used for the control of the virus and bacterial infections [11,12]. However, further functional verification of the relationship between cytosporone B and the putative receptor in this signaling cascade must be studied.

In conclusion, for the first time, we revealed that cytosporone B has a promising effect on the control of citrus decay caused by *G. citri-aurantii* that is comparable to that of the commercialized fungicide prochloraz. In addition, cytosporone B led to a large number of genes changing their expression levels in *G. citri-aurantii*, including 1412 upregulated unigenes and 2128 downregulated unigenes, suggesting that the mode of action of cytosporone B is possibly associated with multiple interacting genes that would be beneficial for counteracting the development of resistance in *G. citri-aurantii*. However, similar to the resistance risk assessment for prochloraz, it is inferred that *G. citri-aurantii* may mutate the genes in the pathway of amino acid and carbon metabolism to increase the resistance toward cytosporone B. Our research provides an essential molecular basis for the application and management of a novel fungicide, cytosporone B.

## Supplementary Materials

The supplementary materials are available online at https://www.mdpi.com/2218-273X/9/4/125/s1.

## Abbreviations

EC_50_	Median effect concentration
MIC	minimum inhibitory concentration
SEM	scanning electron microscopy
PI	propidium iodide
PDA	potato dextrose agar
NR	nonredundant protein database
GO	gene Ontology
COG	Clusters of Orthologous Groups
KEGG	Kyoto Encyclopedia of Genes and Genomes
PCC	pearson’s correlation coefficient
DEGs	differential expression of genes
qRT-PCR	quantitative real-time PCR
