# Supplementary material for: Cytosporone B as a Biological Preservative: Purification, Fungicidal Activity and Mechanism of Action against Geotrichum citri-aurantii
Source: Biomolecules. 2019 Mar 29;9(4):125. doi: 10.3390/biom9040125 (PMC6523523; doi:10.3390/biom9040125)
Supplement: Supplementary file 1 [file biomolecules-09-00125-s001.zip › biomolecules-462950-supplementary final/Supplementary file 1.pdf]

Unigene name	Sequence
c27184-F	5' GGGTTATGGATGATCTTATTGTGC 3'
c27184-R	5' CCGACTTGCCGCTATCTGTAT 3'
c26973-F	5' CTCCTGCTCAATGCCAAACCAAA 3
c26973-R	5' CTGAGACCCGTTGCCATTTACCC 3
c27242-F	5' CTGGTGGGCAAAGTCAAGCAA 3'
c27242-R	5' TCCGCATTGTCAAGAGTAGAAACG 3'
c26832-F	5' TGAATACCGTAGTTCCAACCAATAGC 3'
c26832-R	5' ACAATGGTGGTCCCGCTTCT 3'
c26914-F	5' ACGATACGTTTGAGGCCACAGC 3'
c26914-R	5' CATTACACGGCGACCAACCAC 3'
c22418-F	5' TTCAGGCGGATCGTCGTTT 3'
c22418-R	5' GCAGCATCGCATAGCTCACA 3'
c28014-F	5' GAAGGCGTAAACTGCTCCAACAC 3'
c28014-R	5' AGGGCCGCGACACTAGACAA 3'
c28250-F	5' TATGTGGGTGTCTCAGCAGTCG 3'
c28250-R	5' GGGTTCATTTGGCAAGGTCA 3'
c14704-F	5' TTCCACCACCAACTGTCTCG 3'
c14704-R	5' AATGGTCCCACTGTTGCTGTC 3'
c27638-F	5' ATTGATCACGCTGGTTCG 3’
c27638-R	5’ GACCCTCAACGCTGAGC 3’
Reference gene	Sequence
β-Actin-F:	5' GAGCGGCCAATCTTGACAAAACC 3'
β-Actin-R:	5' CAGCAACCCTCCATTTAGACACCAC 3'
