# Supplementary material for: Cytosporone B as a Biological Preservative: Purification, Fungicidal Activity and Mechanism of Action against Geotrichum citri-aurantii
Source: Biomolecules. 2019 Mar 29;9(4):125. doi: 10.3390/biom9040125 (PMC6523523; doi:10.3390/biom9040125)
Supplement: Supplementary file 1 [file biomolecules-09-00125-s001.zip › biomolecules-462950-supplementary final/supplementary file 2.pdf]

Cytosporone B as a biological preservative: purification, structure identification and mechanism of action against *Geotrichum citri-aurantii*

Chunxiao Yin [a, b,†], Hongxin Liu [c, †], Yang Shan[b], Vijai Kumar Gupta[d,*], Yueming Jiang [a, f], Weimin Zhang[b, *], Haibo Tan[a], Liang Gong [a, f, *]

[a]Key Laboratory of Plant Resource Conservation and Sustainable Utilization, Guangdong Provincial Key Laboratory of Applied Botany, South China Botanical Garden, Chinese Academy of Sciences, Guangzhou 510650, China
[b]Long Ping Branch, Graduate School of Hunan University, Changsha 410125, China
[c]State Key Laboratory of Applied Microbiology Southern China, Guangdong Provincial Key Laboratory of Microbial Culture Collection and Application, Guangdong Open Laboratory of Applied Microbiology, Guangdong Institute of Microbiology, Guangzhou 510070, China
[d]Public Research Laboratory, Hainan Medical University, Haikou 571199, China
[e]School of Life Science, Jiaying University, Meizhou 514015, China
[f]Key Laboratory of Post-Harvest Handling of fruits, Ministry of Agriculture, Guangzhou 510650, China

Contents

Figure S1. ^{1}H NMR spectrum (500 MHz, $CDCl_3$) of **1**....S3

Figure S2. ^{13}C NMR spectrum (125 MHz, $CDCl_3$) of **1**....S4

Figure S3. ^{1}H NMR spectrum (500 MHz, $CDCl_3$) of **2**....S5

Figure S4. ^{13}C NMR spectrum (125 MHz, $CDCl_3$) of **2**....S6

Figure S5. ^{1}H NMR spectrum (500 MHz, $CDCl_3$) of **3**....S7

Figure S6. ^{13}C NMR spectrum (125 MHz, $CDCl_3$) of **3**....S8

Figure S7. ^{1}H NMR spectrum (500 MHz, $CDCl_3$) of **4**....S9

Figure S8. ^{13}C NMR spectrum (125 MHz, $CDCl_3$) of **4**....S10

Figure S9. ^{1}H NMR spectrum (500 MHz, $CDCl_3$) of **5**....S11

Figure S10. ^{13}C NMR spectrum (125 MHz, $CDCl_3$) of **5**....S12

Figure S11. ^{1}H NMR spectrum (500 MHz, $CDCl_3$) of **6**....S13

Figure S12. ^{13}C NMR spectrum (125 MHz, $CDCl_3$) of **6**....S14

Figure S13. ^{1}H NMR spectrum (500 MHz, $CDCl_3$) of **7**....S15

Figure S14. ^{13}C NMR spectrum (125 MHz, $CDCl_3$) of **7**....S16

Figure S15. ^{1}H NMR spectrum (500 MHz, $CDCl_3$) of **8**....S17

Figure S16. ^{13}C NMR spectrum (125 MHz, $CDCl_3$) of **8**....S18

Figure S17. ^{1}H NMR spectrum (500 MHz, $CDCl_3$) of **9**....S19

Figure S18. ^{13}C NMR spectrum (125 MHz, $CDCl_3$) of **9**....S20

Figure S19. ^{1}H NMR spectrum (500 MHz, $CDCl_3$) of **10**....S21

Figure S20. ^{13}C NMR spectrum (125 MHz, $CDCl_3$) of **10**....S22

Figure S21. ^{1}H NMR spectrum (500 MHz, $CDCl_3$) of **11**....S23

Figure S22. ^{13}C NMR spectrum (125 MHz, $CDCl_3$) of **11**....S24

Figure S23. ^{1}H NMR spectrum (500 MHz, $CDCl_3$) of **12**....S25

Figure S24. ^{1}C NMR spectrum (125 MHz, $CDCl_3$) of **12**....S25

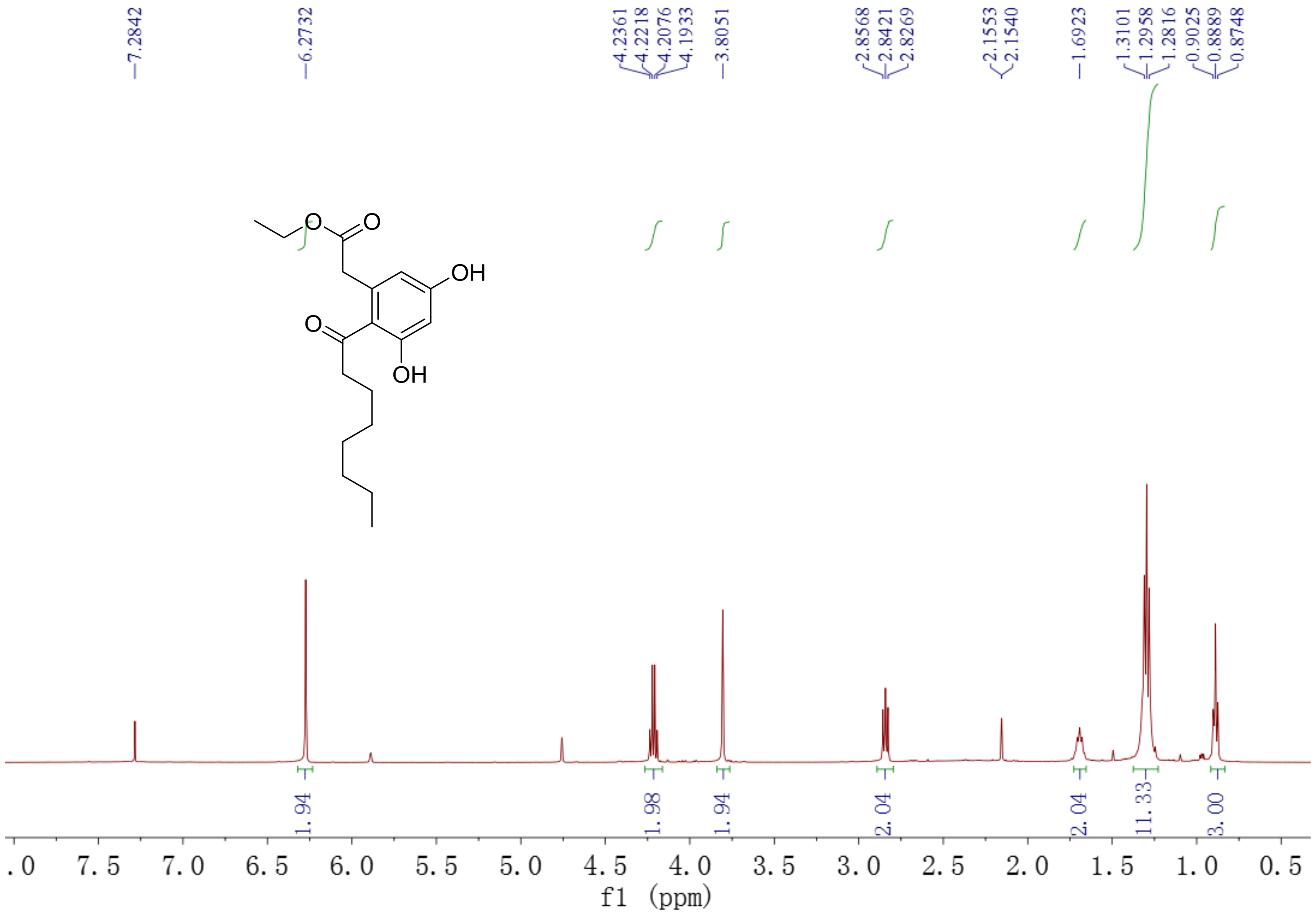

Figure S1. ^{1}H NMR spectrum (500 MHz, $CDCl_3$) of **1**.

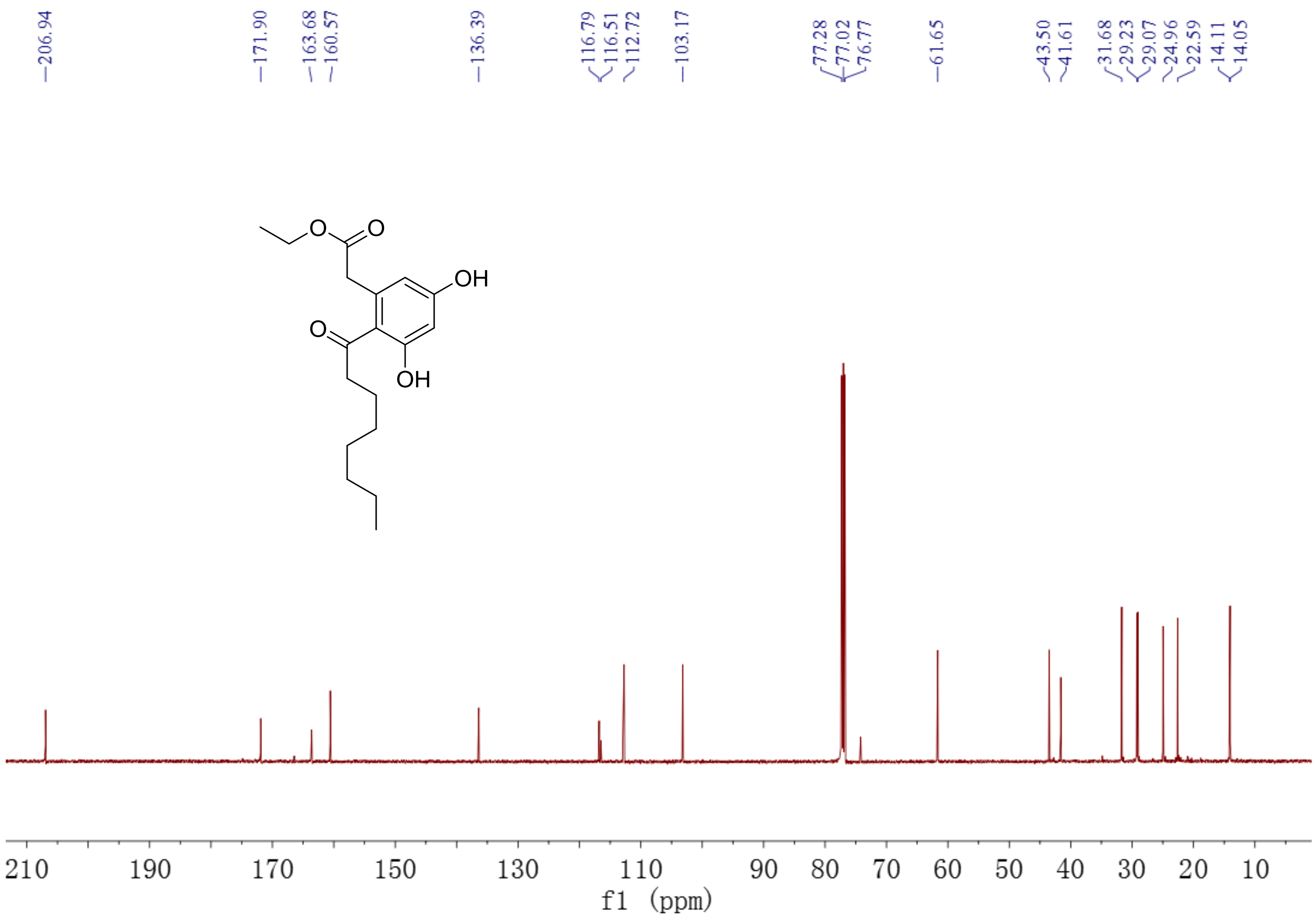

Figure S2. ^{13}C NMR spectrum (125 MHz, $CDCl_3$) of **1**.

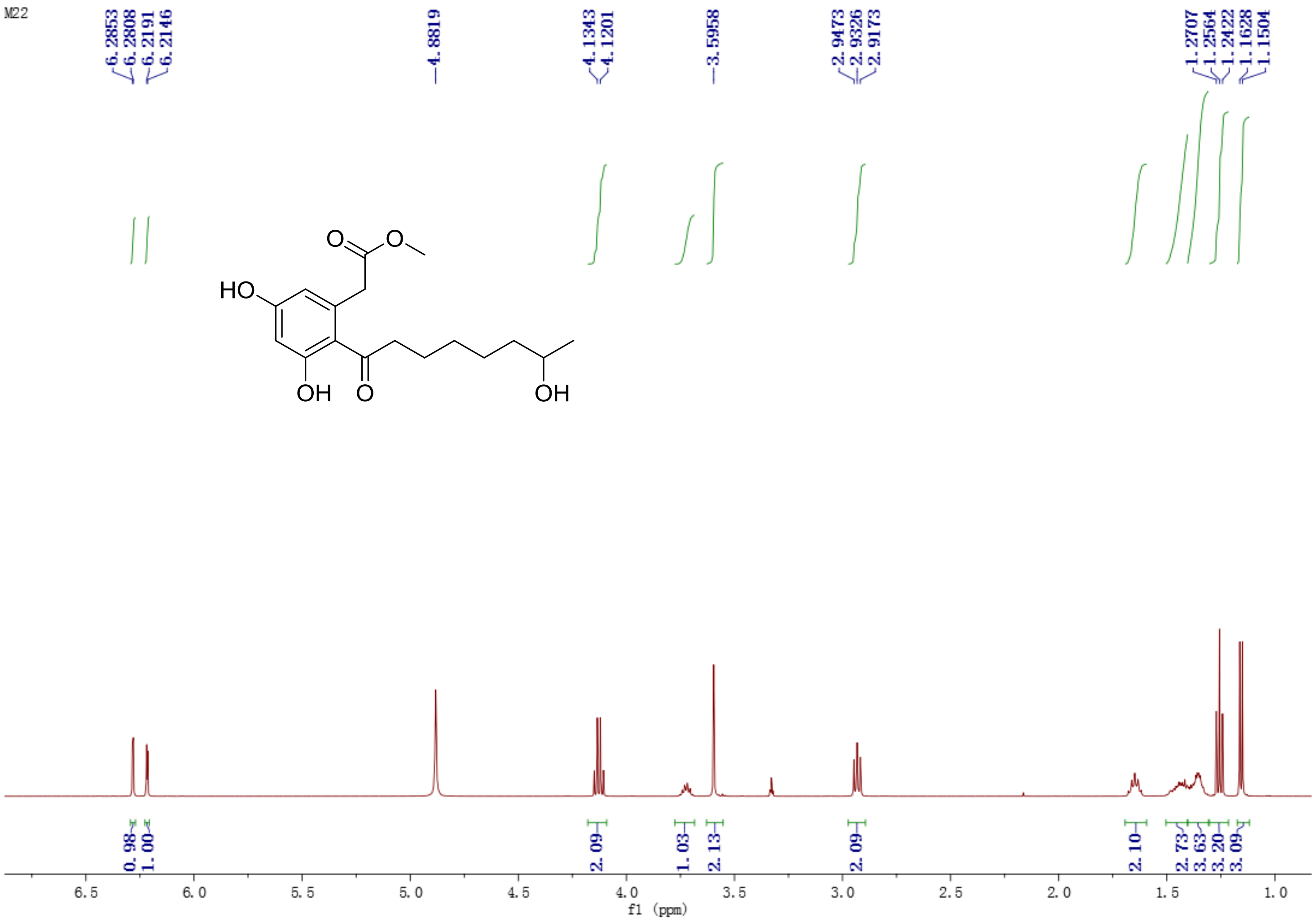

Figure S3. ^{1}H NMR spectrum (500 MHz, $CDCl_3$) of **2**.

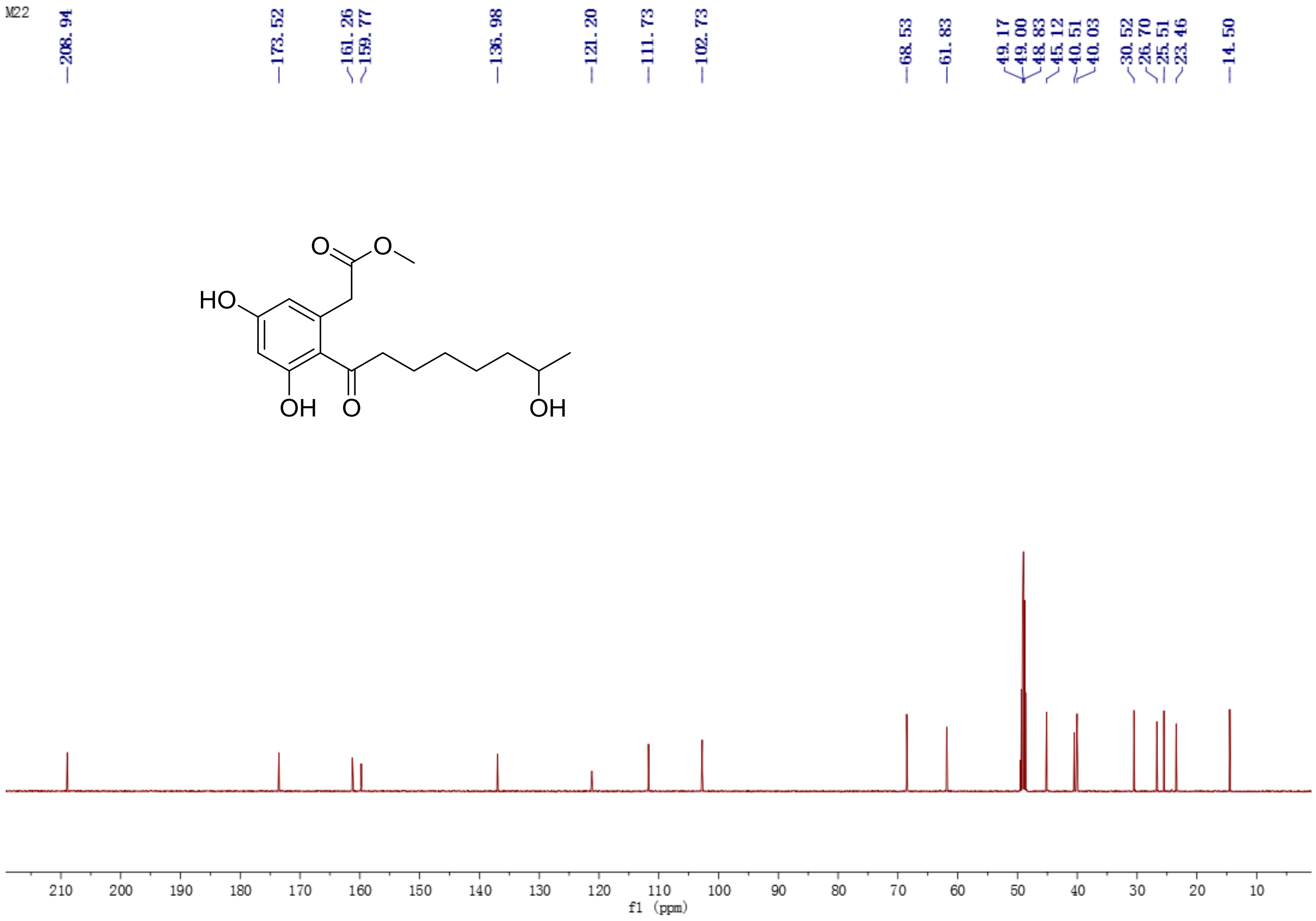

Figure S4. ^{13}C NMR spectrum (125 MHz, $CDCl_3$) of **2**.

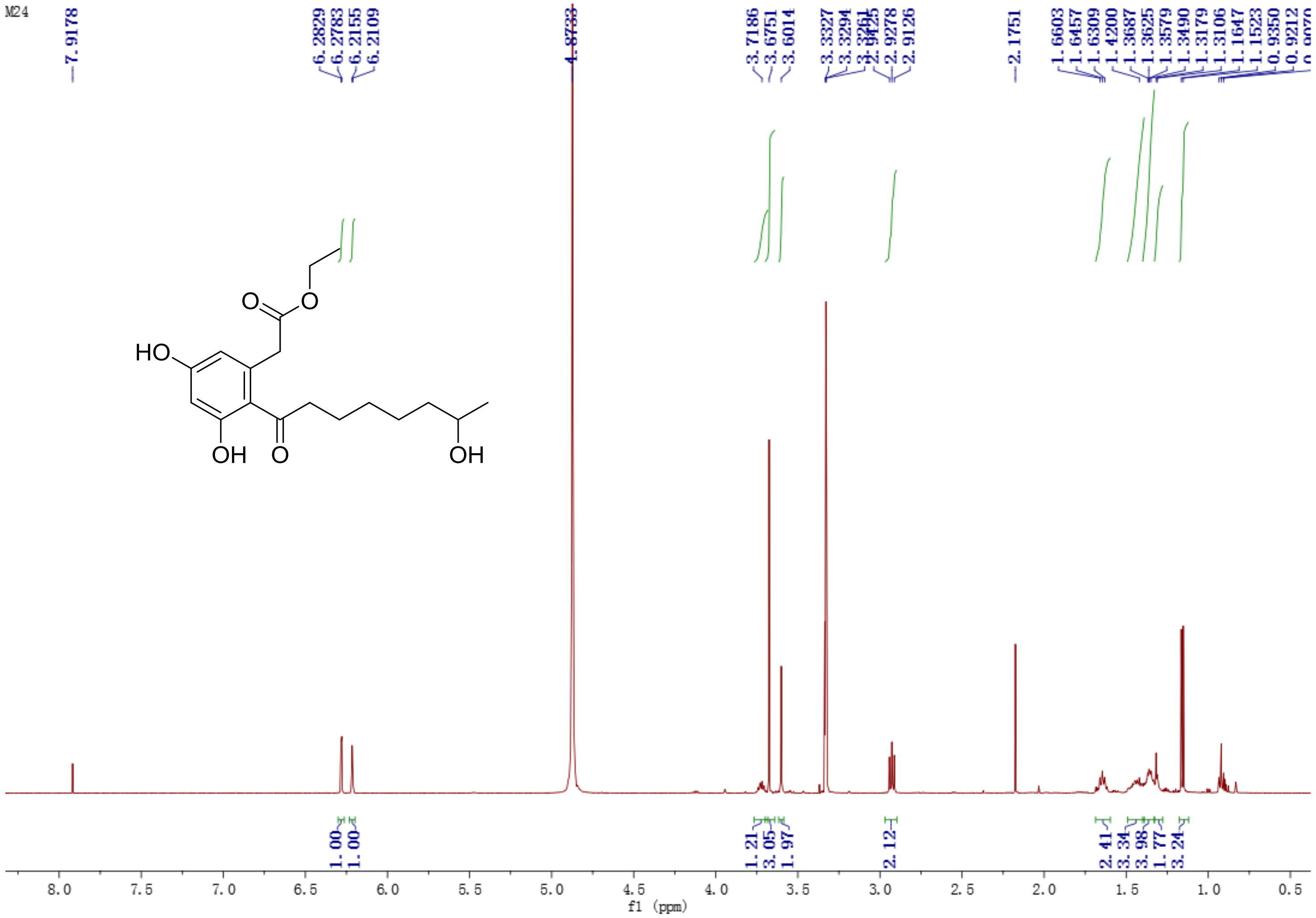

Figure S5. ^{1}H NMR spectrum (500 MHz, $CDCl_3$) of **3**.

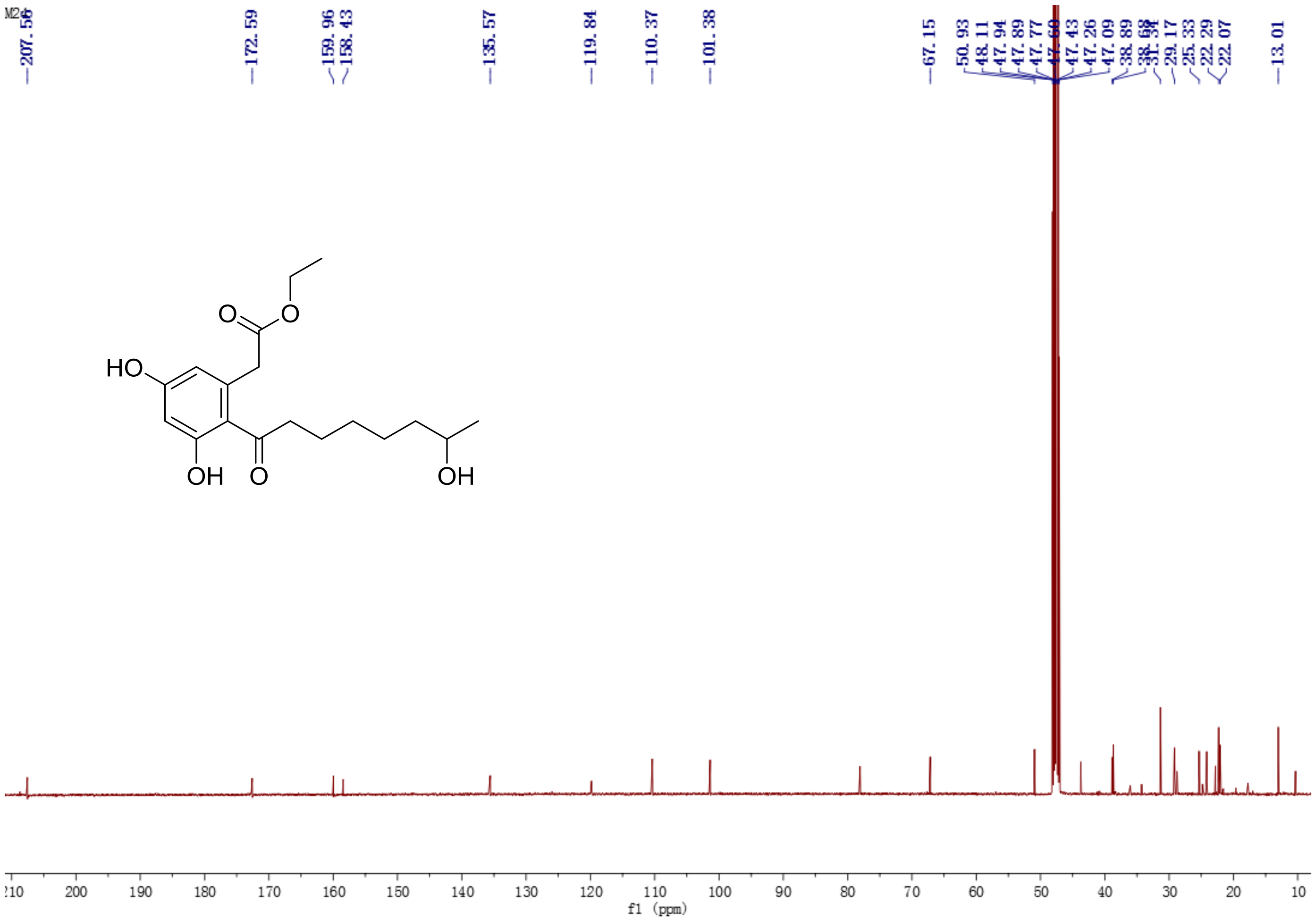

Figure S6. ^{13}C NMR spectrum (125 MHz, $CDCl_3$) of **3**.

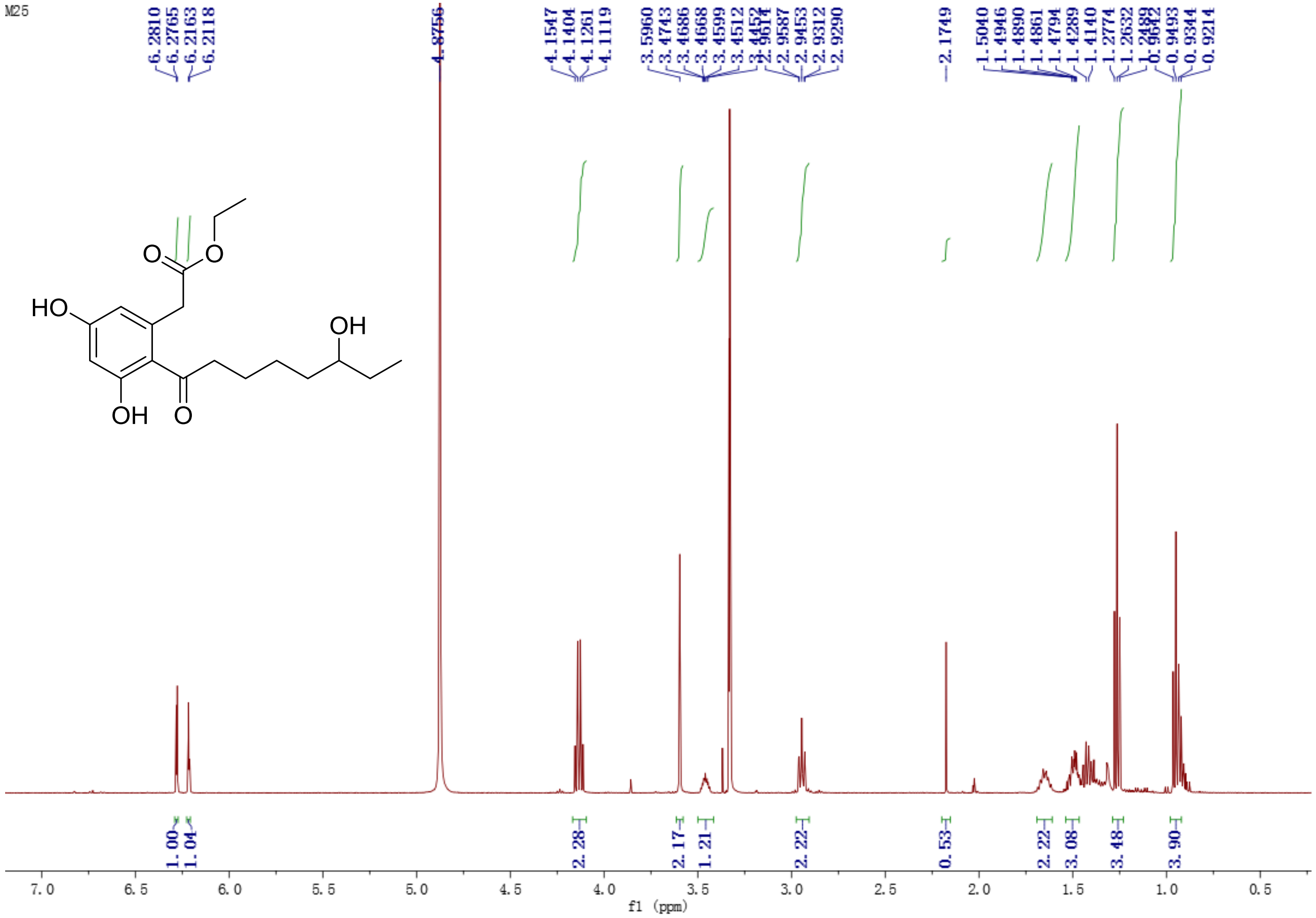

Figure S7. ^{1}H NMR spectrum (500 MHz, $CDCl_3$) of **4**.

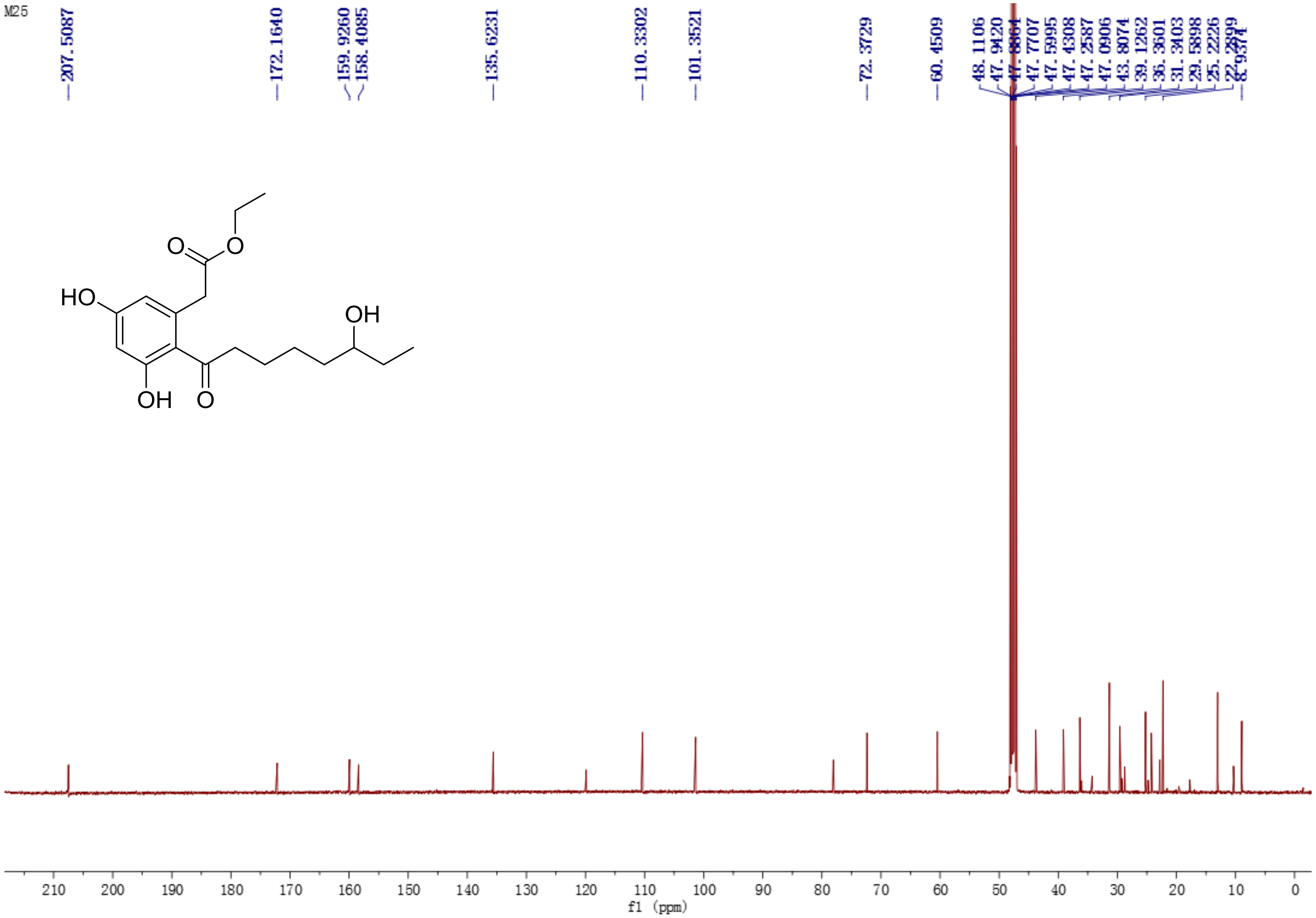

Figure S8. ^{13}C NMR spectrum (125 MHz, $CDCl_3$) of **4**.

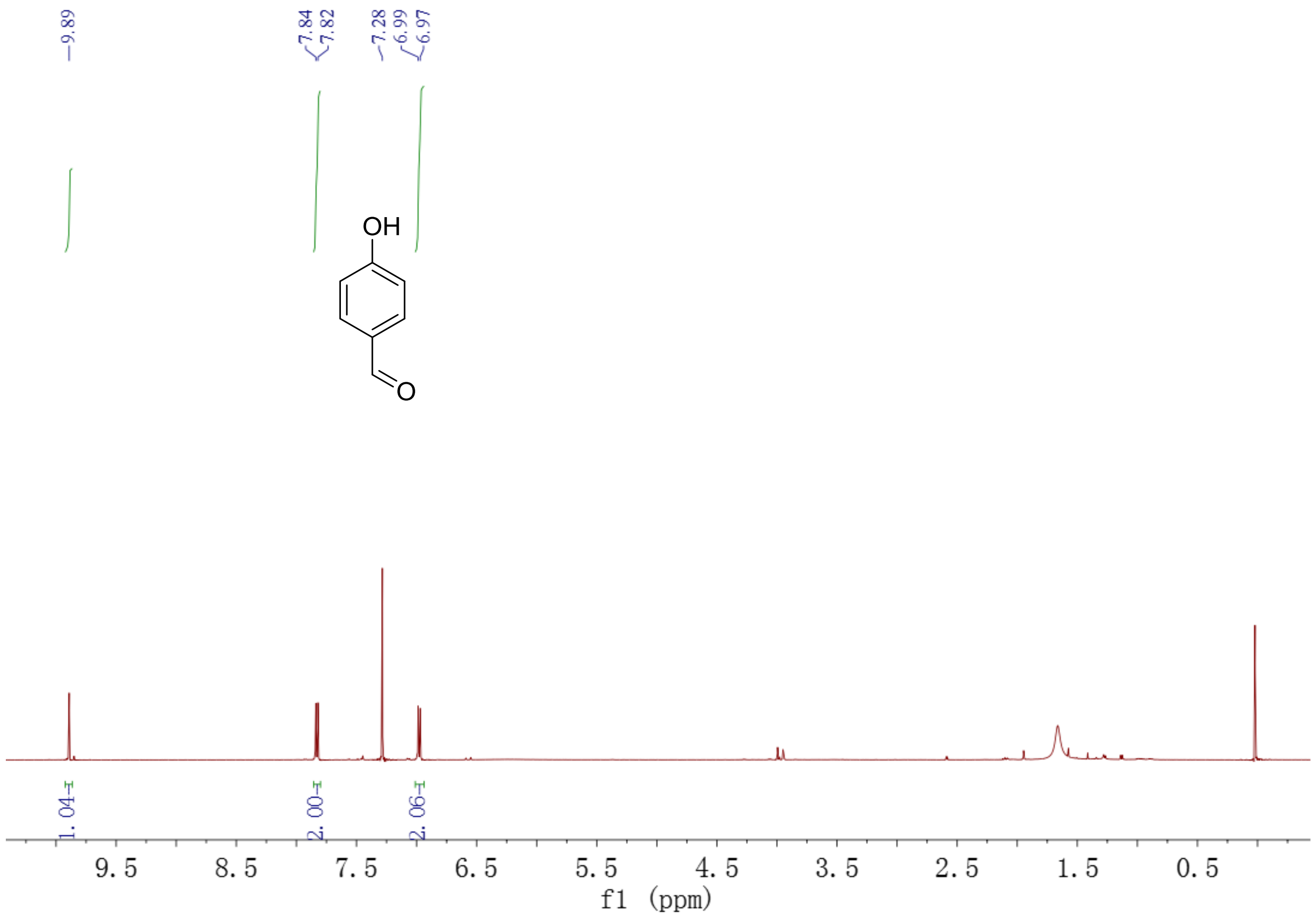

Figure S9. ^{1}H NMR spectrum (500 MHz, $CDCl_3$) of **5**.

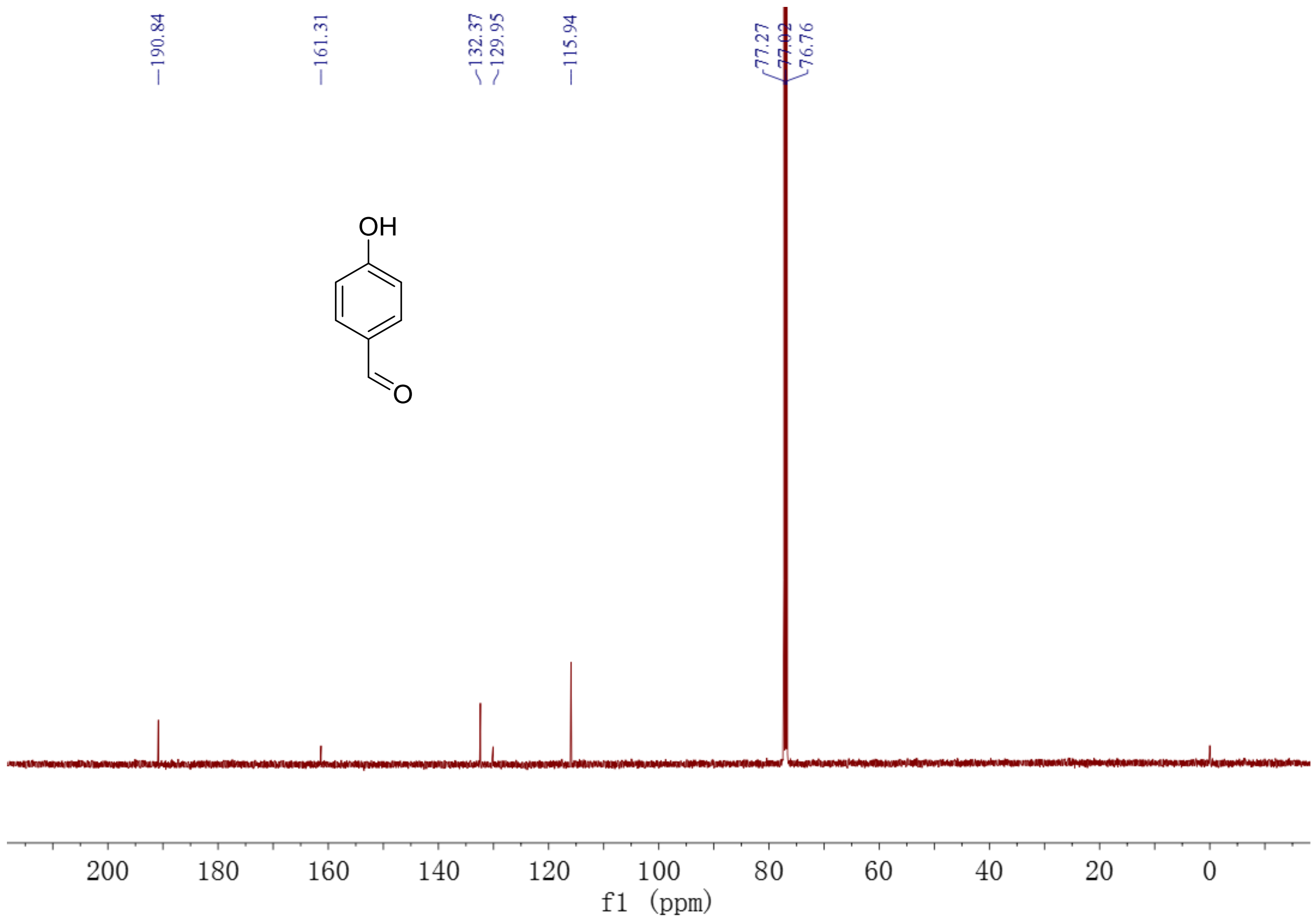

Figure S10. ^{13}C NMR spectrum (125 MHz, $CDCl_3$) of **5**.

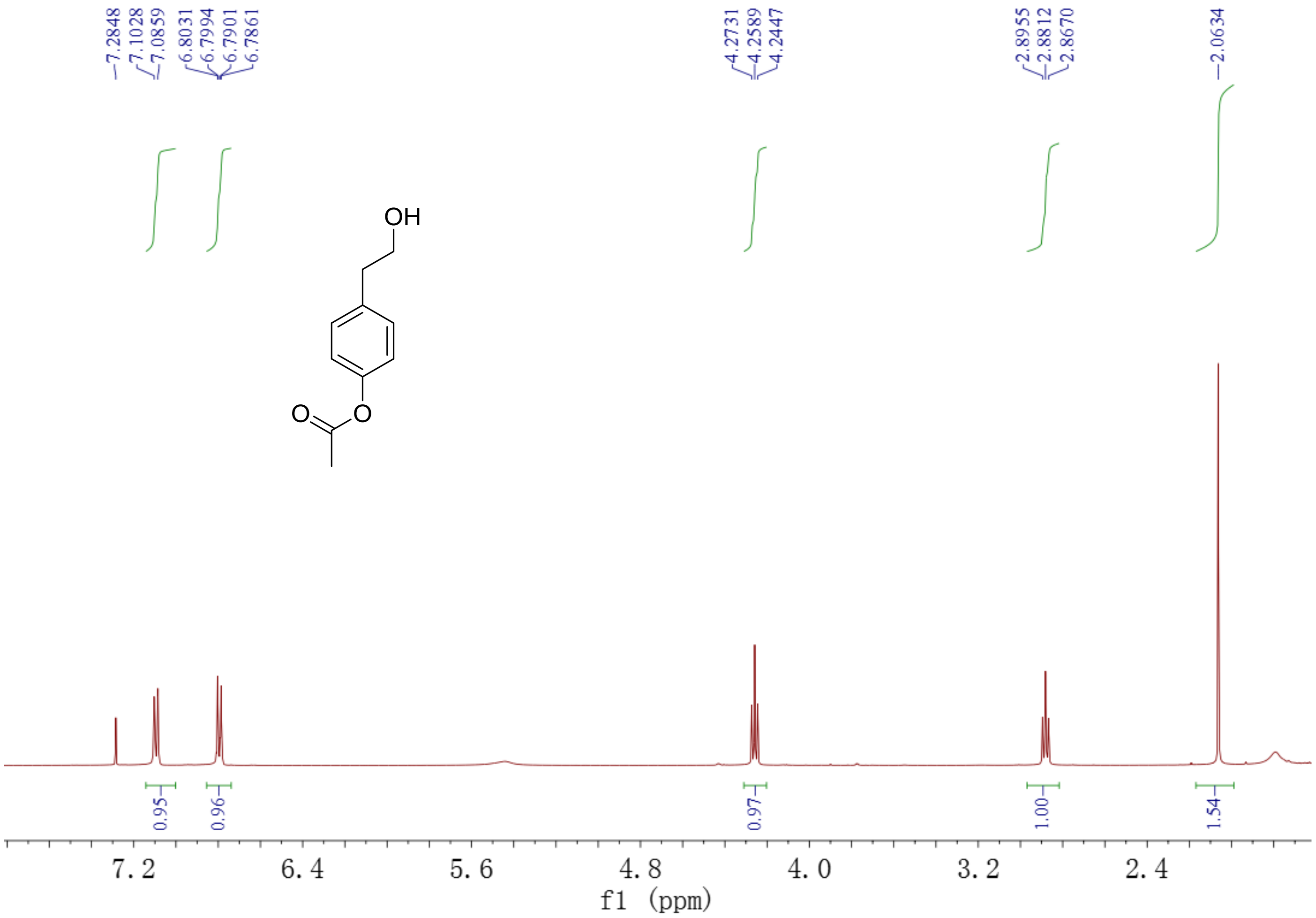

Figure S11. ^{1}H NMR spectrum (500 MHz, $CDCl_3$) of **6**.

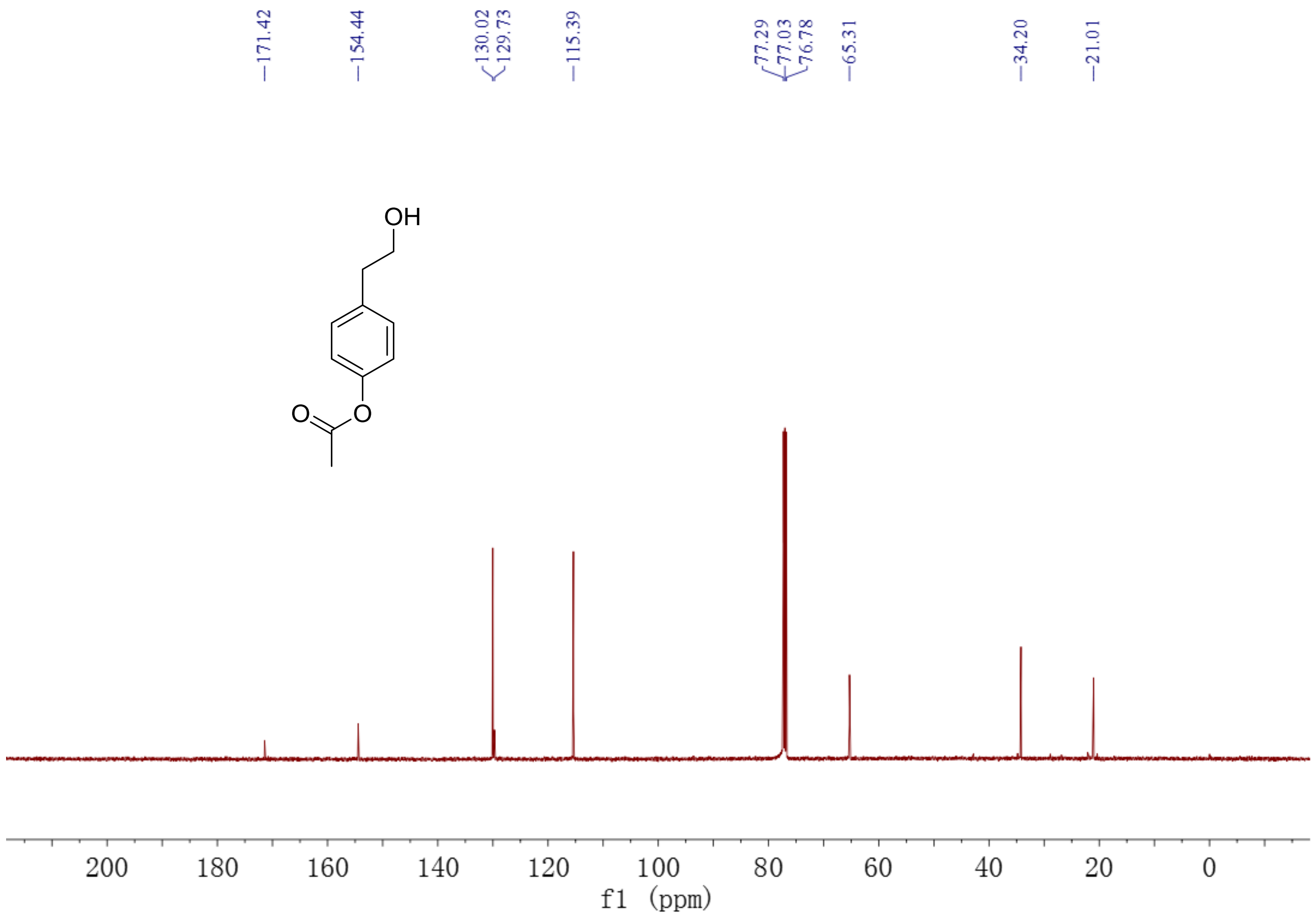

Figure S12. ^{13}C NMR spectrum (125 MHz, $CDCl_3$) of **6**.

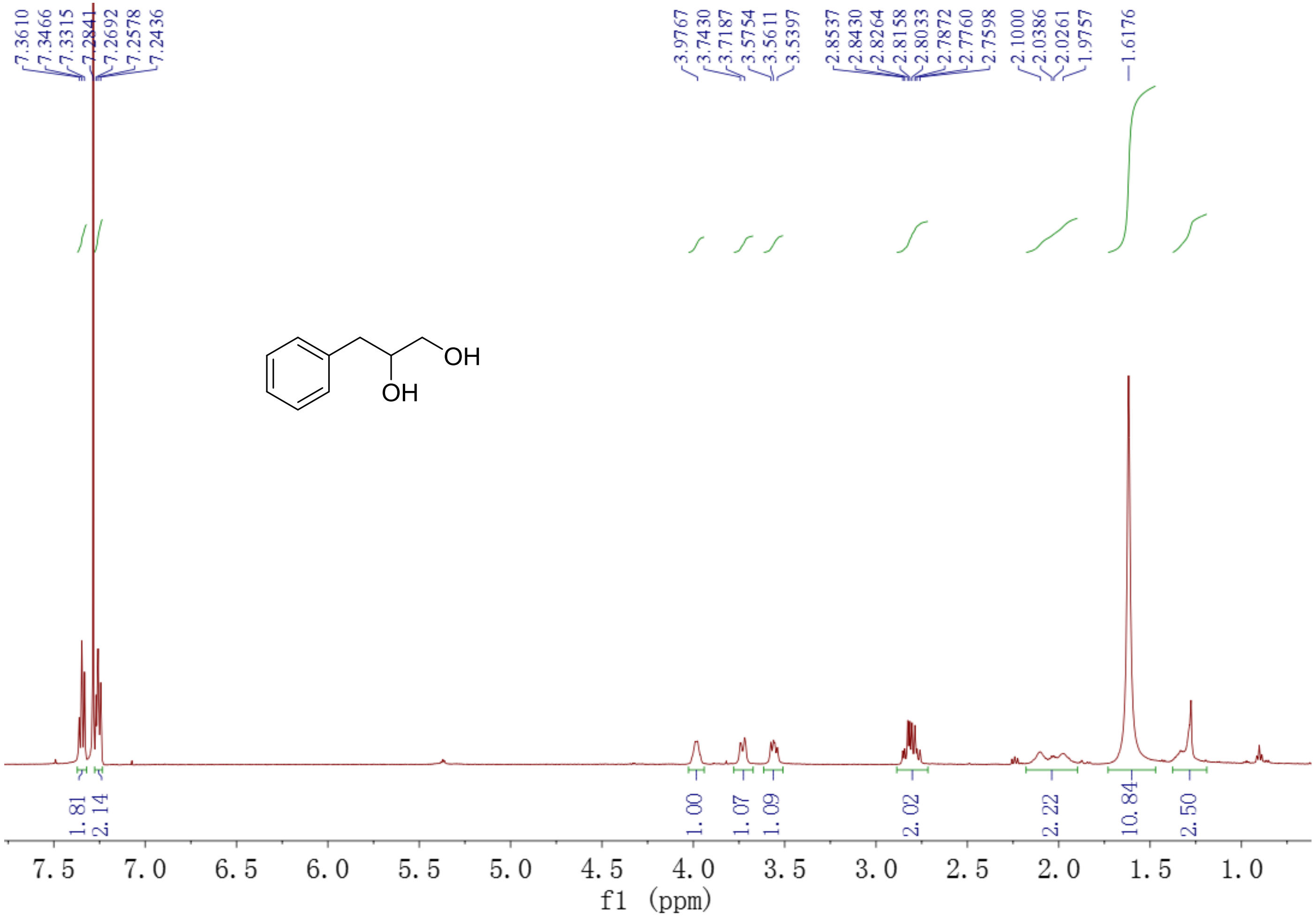

Figure S13. ^{1}H NMR spectrum (500 MHz, $CDCl_3$) of **7**.

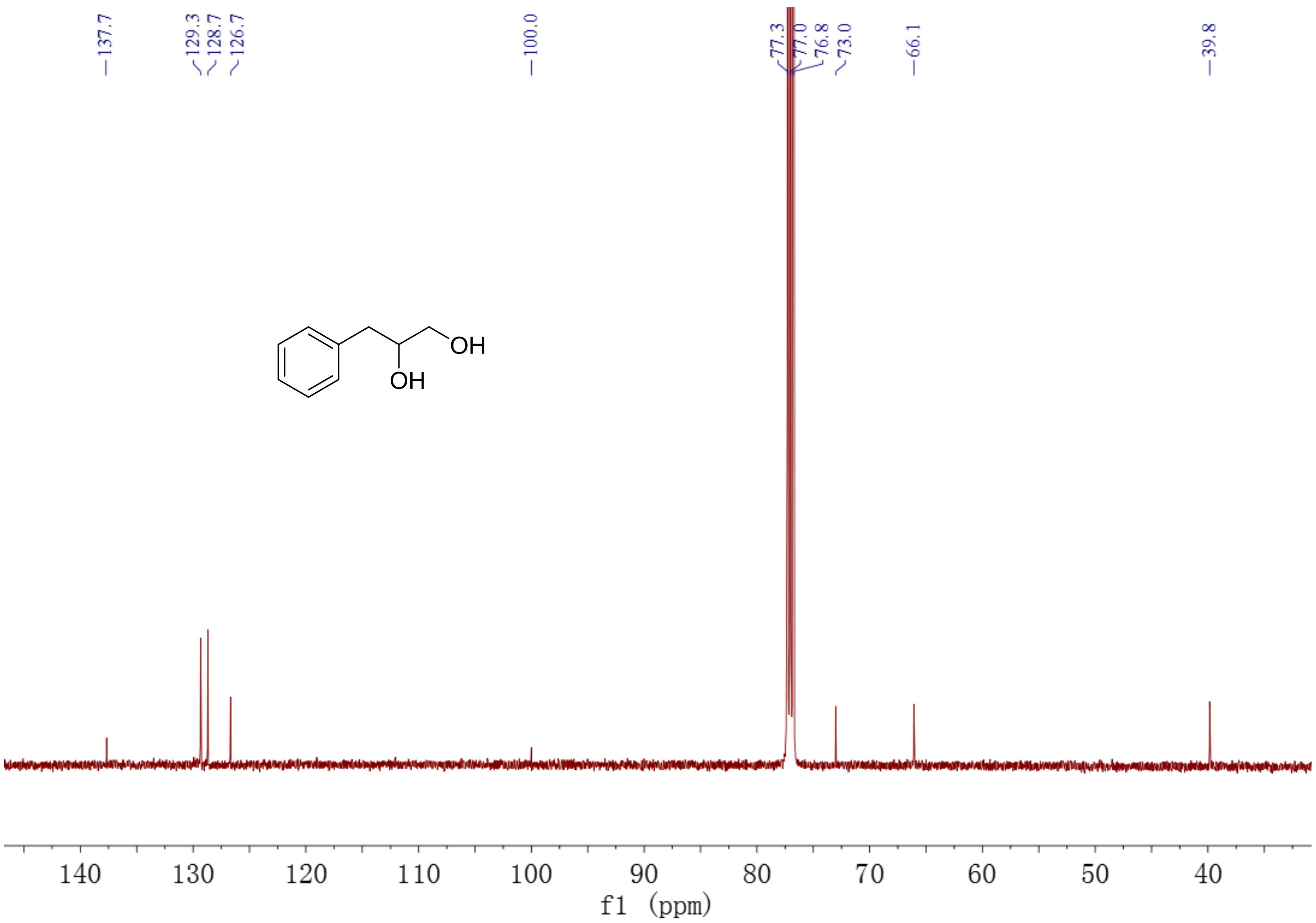

Figure S14. ^{13}C NMR spectrum (125 MHz, $CDCl_3$) of **7**.

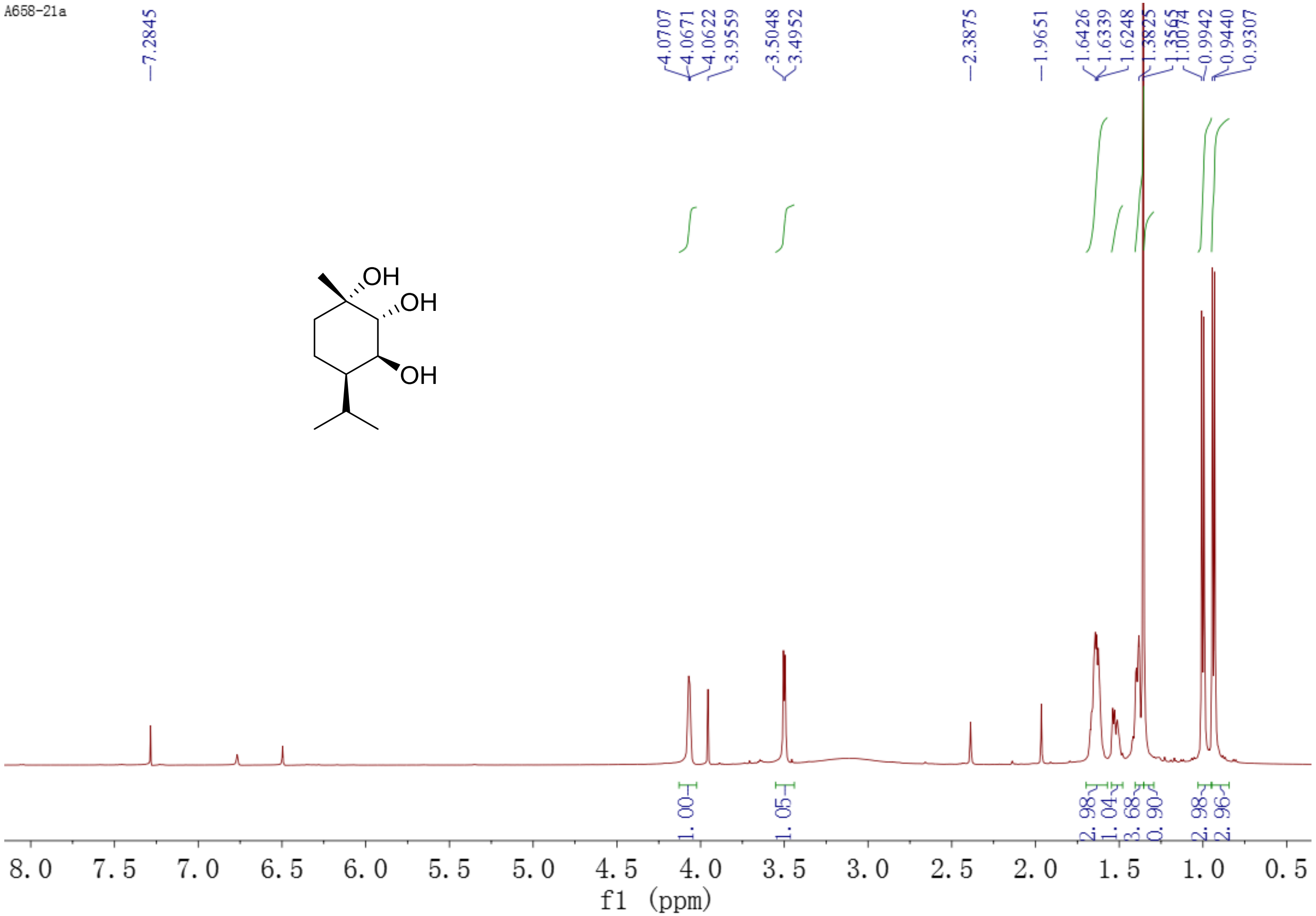

Figure S15. ^{1}H NMR spectrum (500 MHz, $CDCl_3$) of **8**.

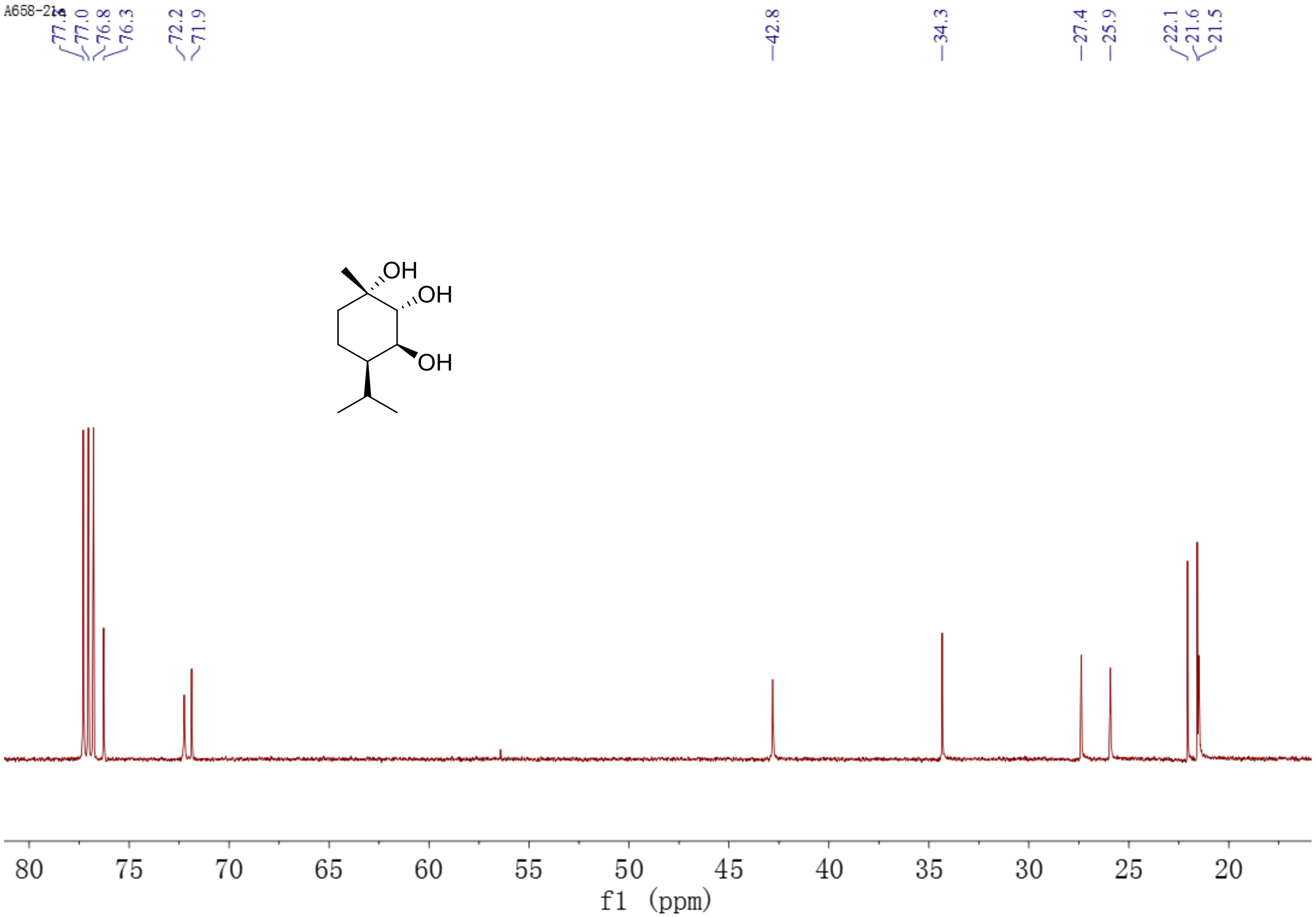

Figure S16. ^{13}C NMR spectrum (125 MHz, $CDCl_3$) of **8**.

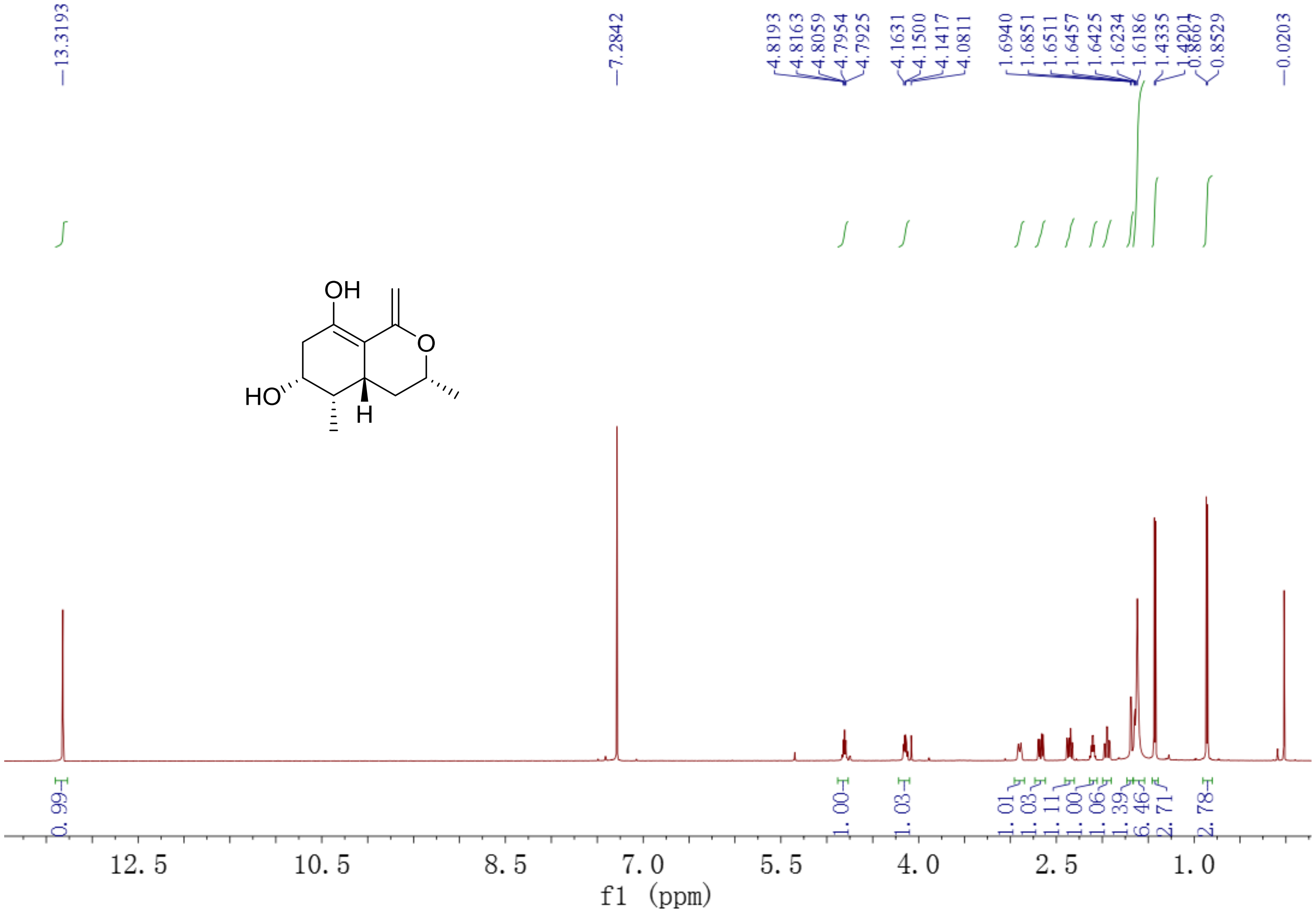

Figure S17. ^{1}H NMR spectrum (500 MHz, $CDCl_3$) of **9**.

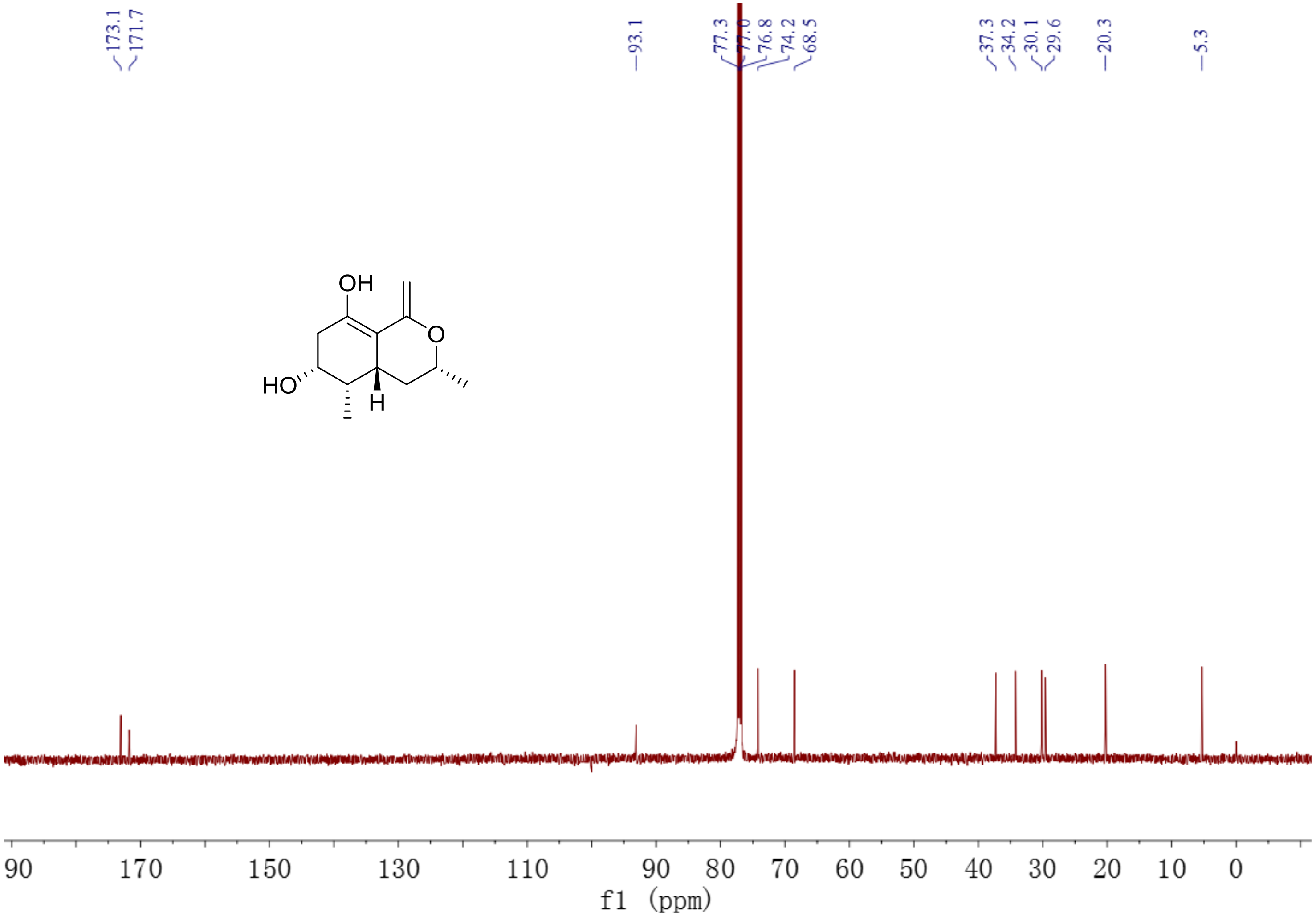

Figure S18. ^{13}C NMR spectrum (125 MHz, $CDCl_3$) of **9**.

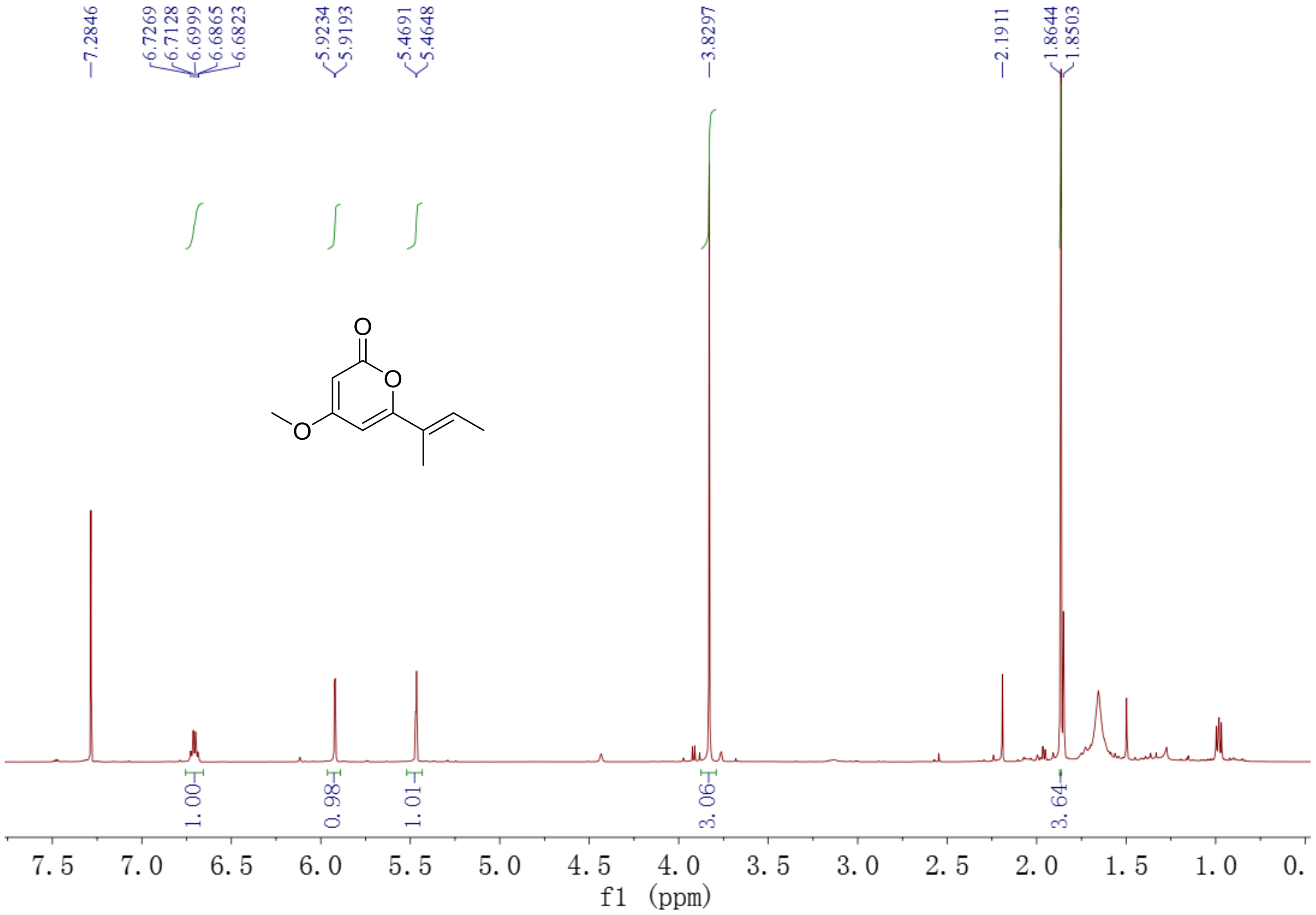

Figure S19. ^{1}H NMR spectrum (500 MHz, $CDCl_3$) of **10**.

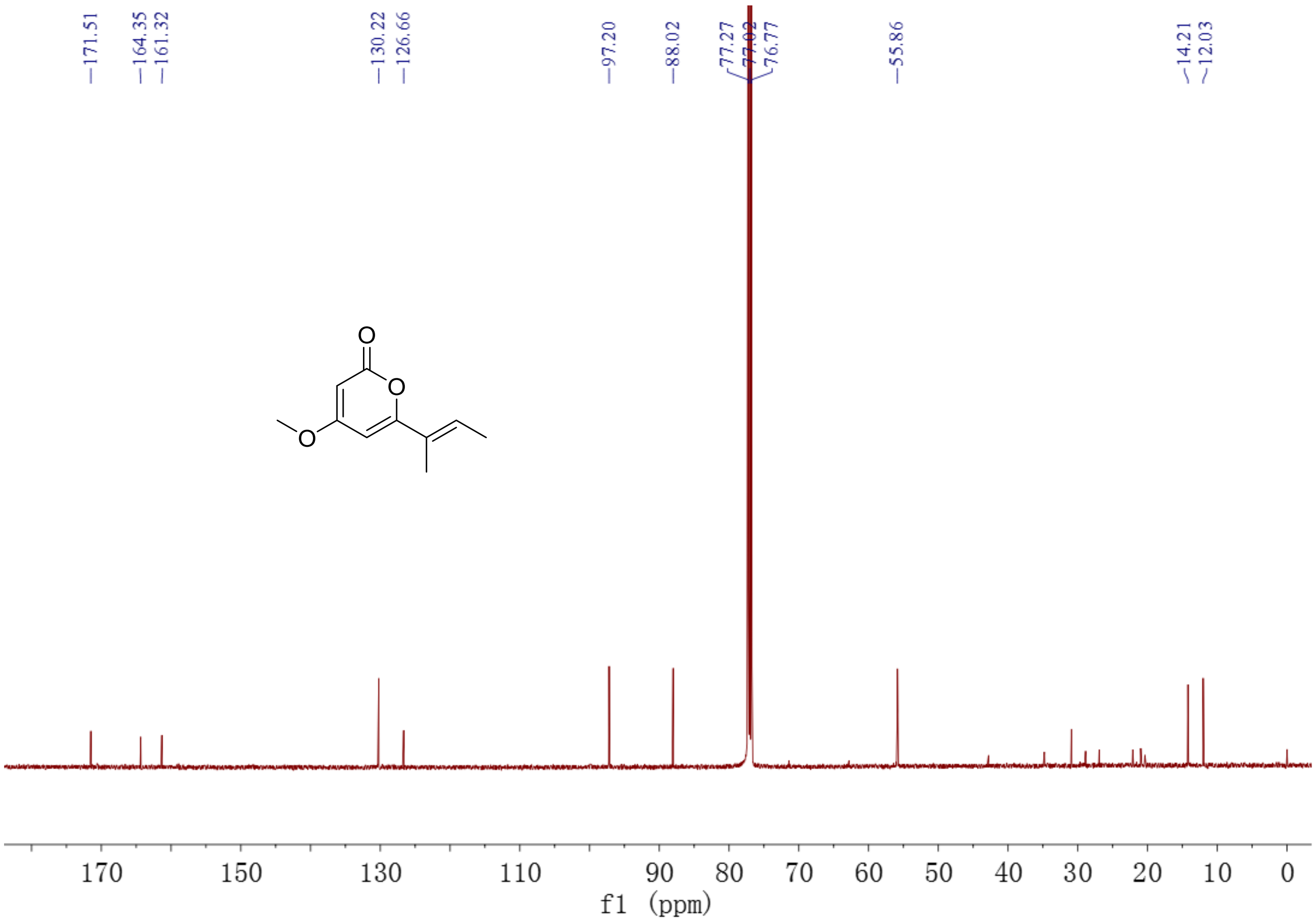

Figure S20. ^{13}C NMR spectrum (125 MHz, $CDCl_3$) of **10**.

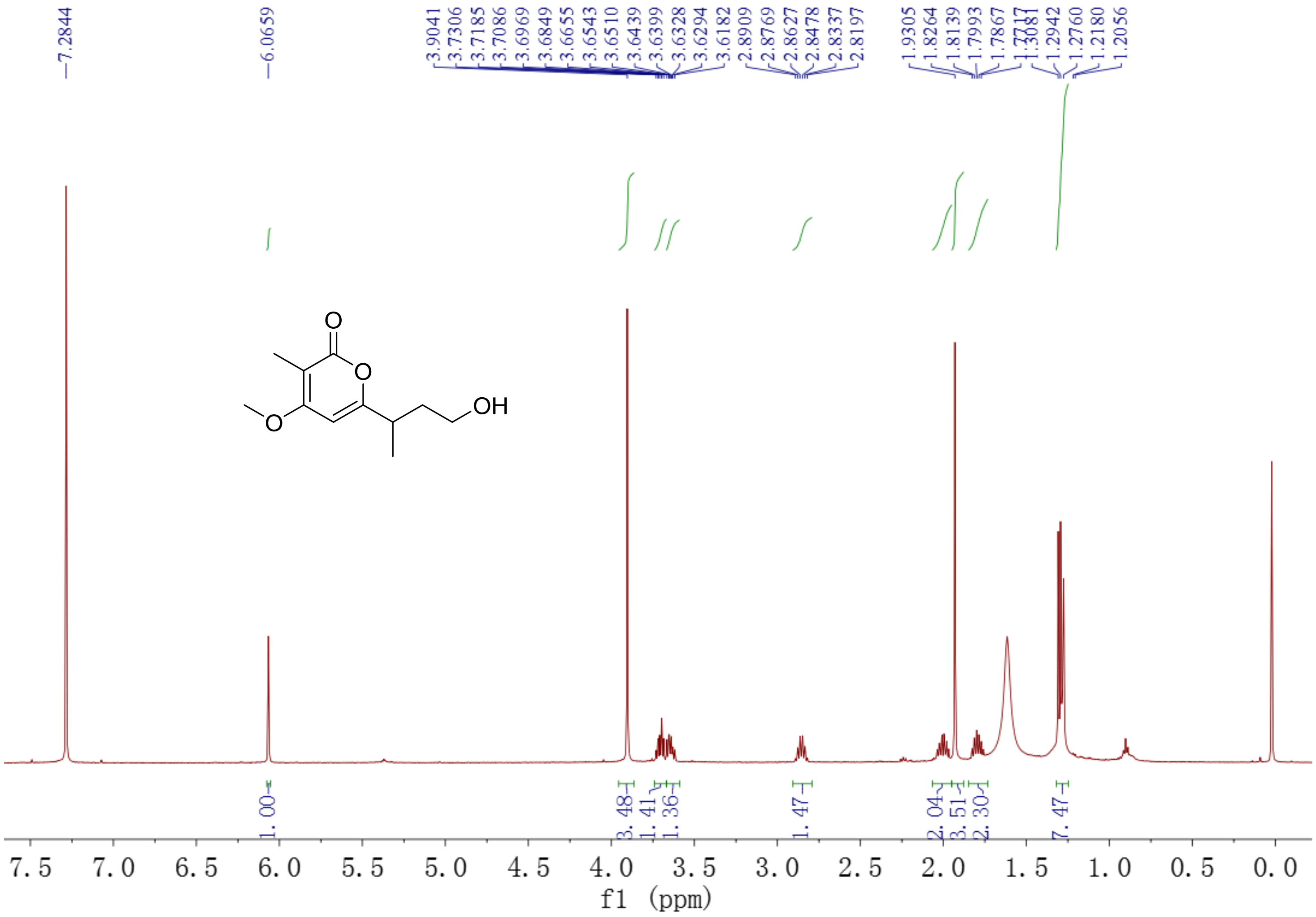

Figure S21. ^{1}H NMR spectrum (500 MHz, $CDCl_3$) of **11**.

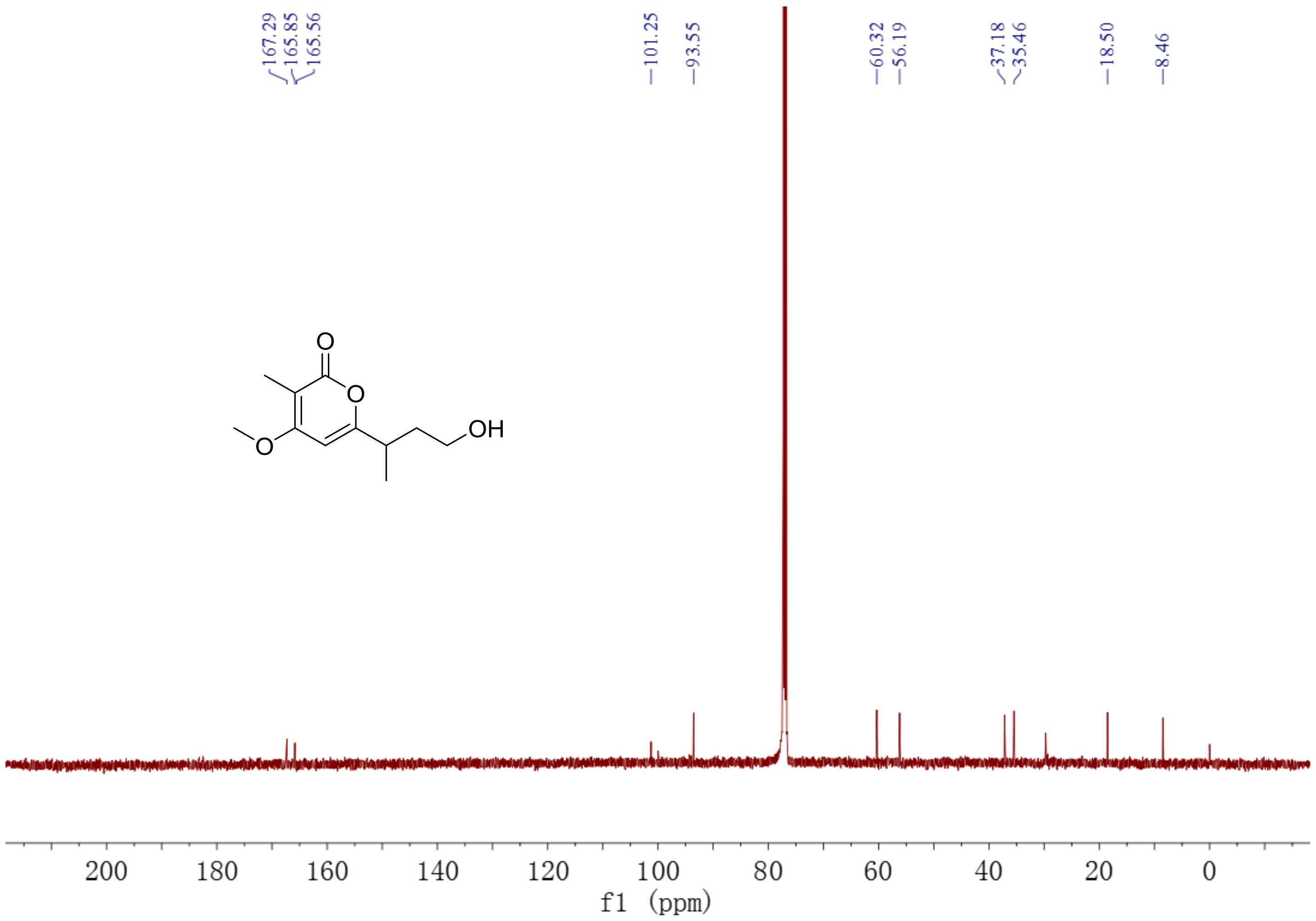

Figure S22. ^{13}C NMR spectrum (125 MHz, $CDCl_3$) of **11**.

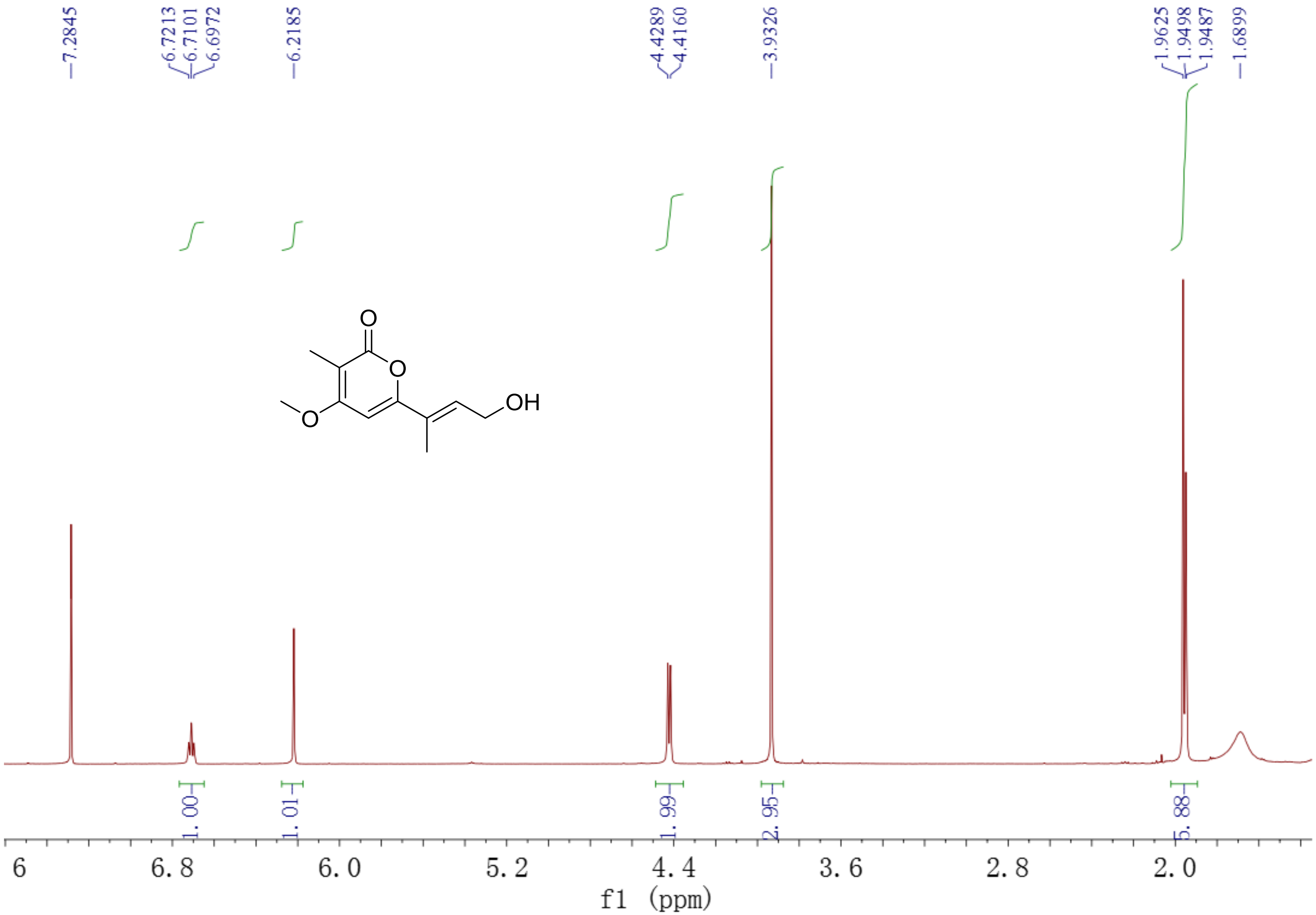

Figure S23. ^{1}H NMR spectrum (500 MHz, $CDCl_3$) of **12**.

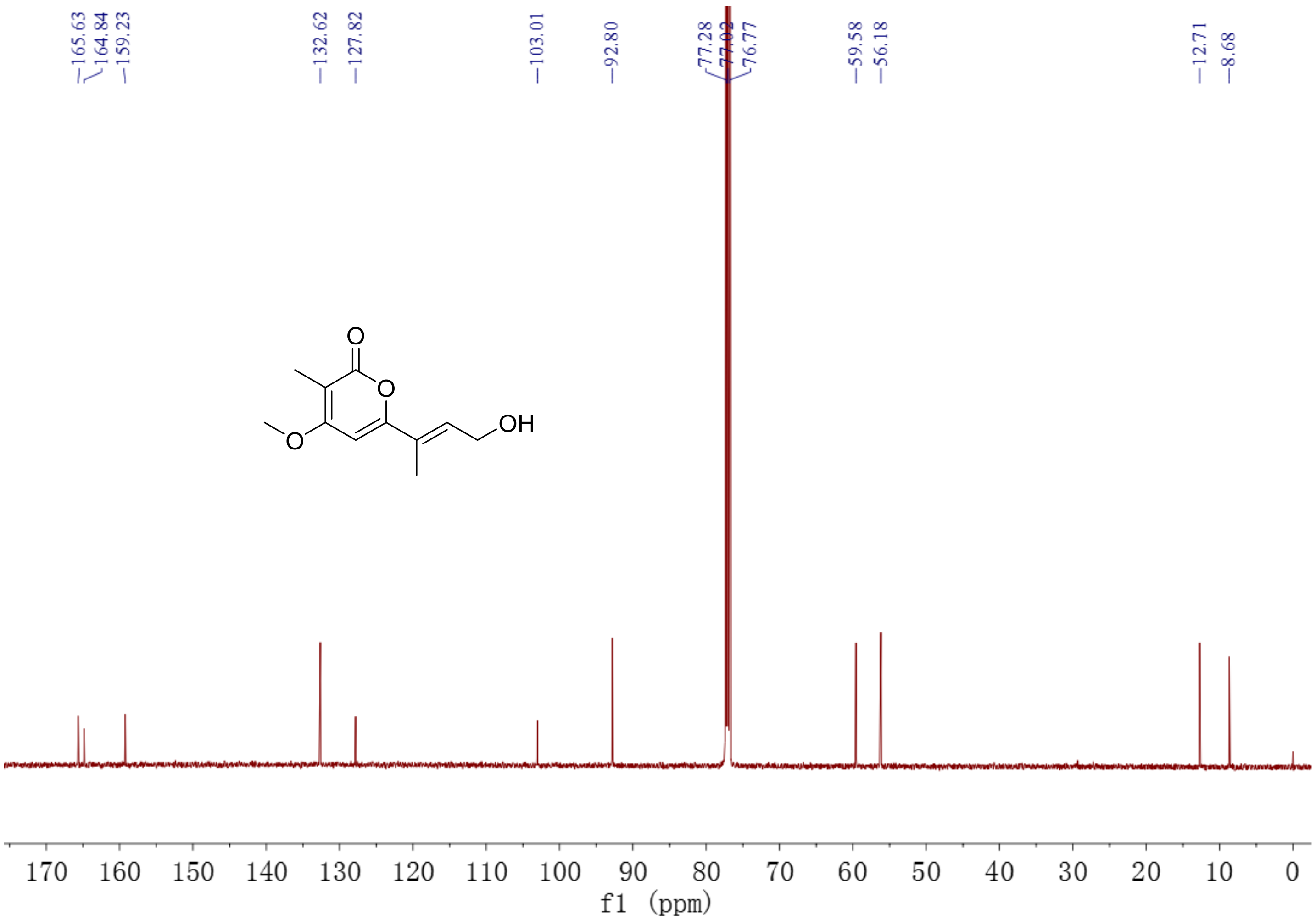

Figure S24. ^{13}C NMR spectrum (125 MHz, $CDCl_3$) of **12**.
