# Supplementary material for: Cytosporone B as a Biological Preservative: Purification, Fungicidal Activity and Mechanism of Action against Geotrichum citri-aurantii
Source: Biomolecules. 2019 Mar 29;9(4):125. doi: 10.3390/biom9040125 (PMC6523523; doi:10.3390/biom9040125)
Supplement: Supplementary file 1 [file biomolecules-09-00125-s001.zip › biomolecules-462950-supplementary final/Supplementary file 3.pdf]

Table S1: Raw data and quality analysis

Samples	Read Number	Base Number	GC Content	Q30
CK1	21,635,277	6,472,224,836	49.96%	87.38%
CK2	27,442,858	8,219,515,664	49.80%	87.14%
CK3	28,500,163	8,523,530,860	49.95%	86.94%
T1	24,109,625	7,214,171,494	49.02%	93.45%
T2	24,869,729	7,434,822,846	48.79%	93.63%
T3	23,856,426	7,149,200,002	49.82%	87.23%

Table S2: Length range of the unigenes

Length Range	Unigene
200-300	23,992(42.91%)
300-500	13,939(24.93%)
500-1000	9,217(16.49%)
1000-2000	5,136(9.19%)
2000+	3,622(6.48%)
Total Number	55906
Total Length	37227738
N50 Length	1130

Mean Length	665.9

Table S3: Annotation of the unigenes

Anno_Database	Annotated_Number	300<=length<1000	length>=1000
COG_Annotation	11240	4612	3562
GO_Annotation	24611	10781	4415
KEGG_Annotation	10843	4400	3675
KOG_Annotation	22067	9446	5294
Pfam_Annotation	22222	9565	6656
Swissprot_Annotation	13471	5140	5388
eggNOG_Annotation	35362	15306	7191
nr_Annotation	36964	15850	7296
All_Annotated	37302	15948	7380

Nr(NCBI nonredundant protein databases); COG (Clusters of Orthologous Groups); KOG (euKaryotic Orthologous Groups); GO(Gene Ontology); KEGG (Kyoto Encyclopedia of Genes and Genomes); Pfam (Protein family).

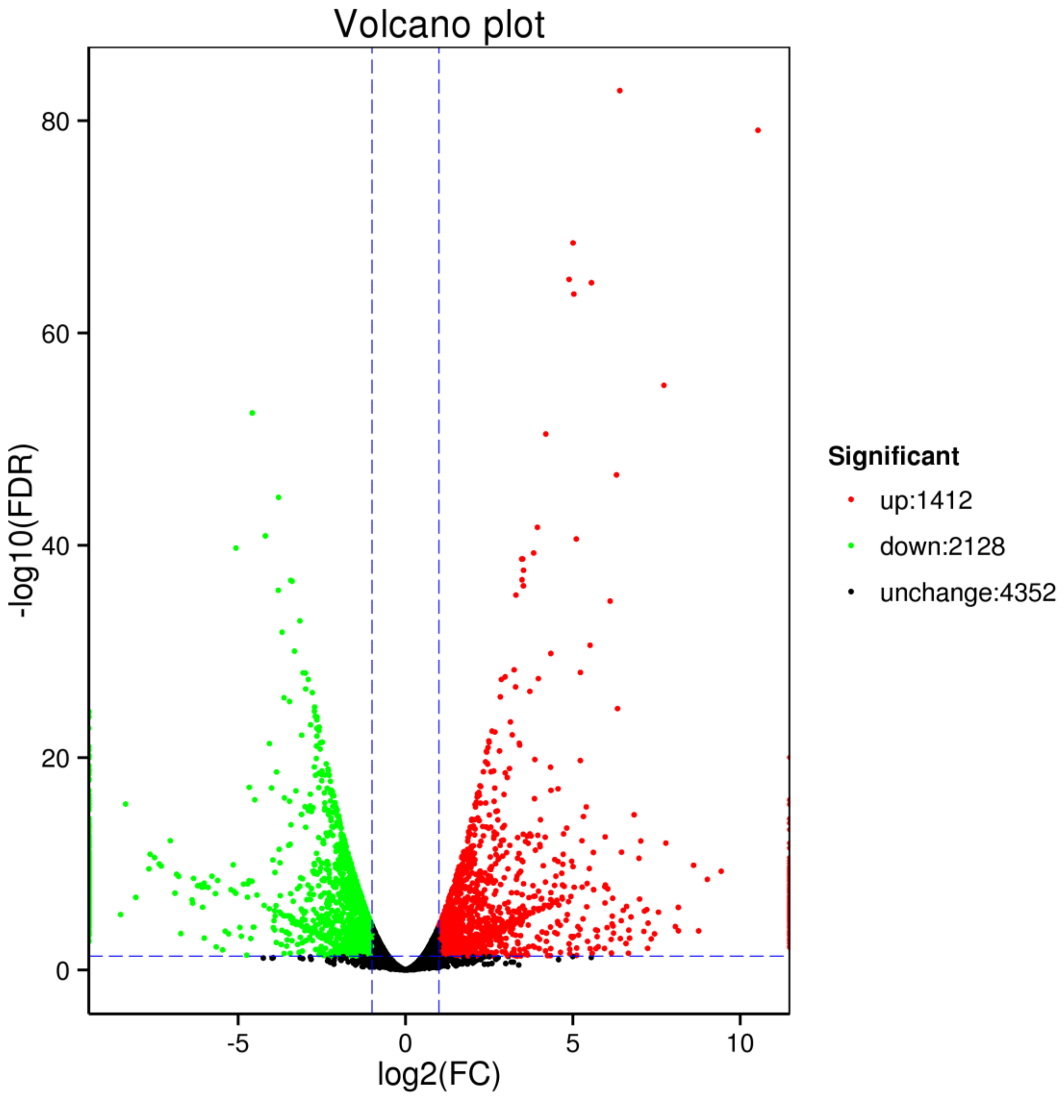

Figure S1: Volcano plot analyses of differentially expressed genes in *G. citri-aurantii* underlying the treatment of cytosporone B

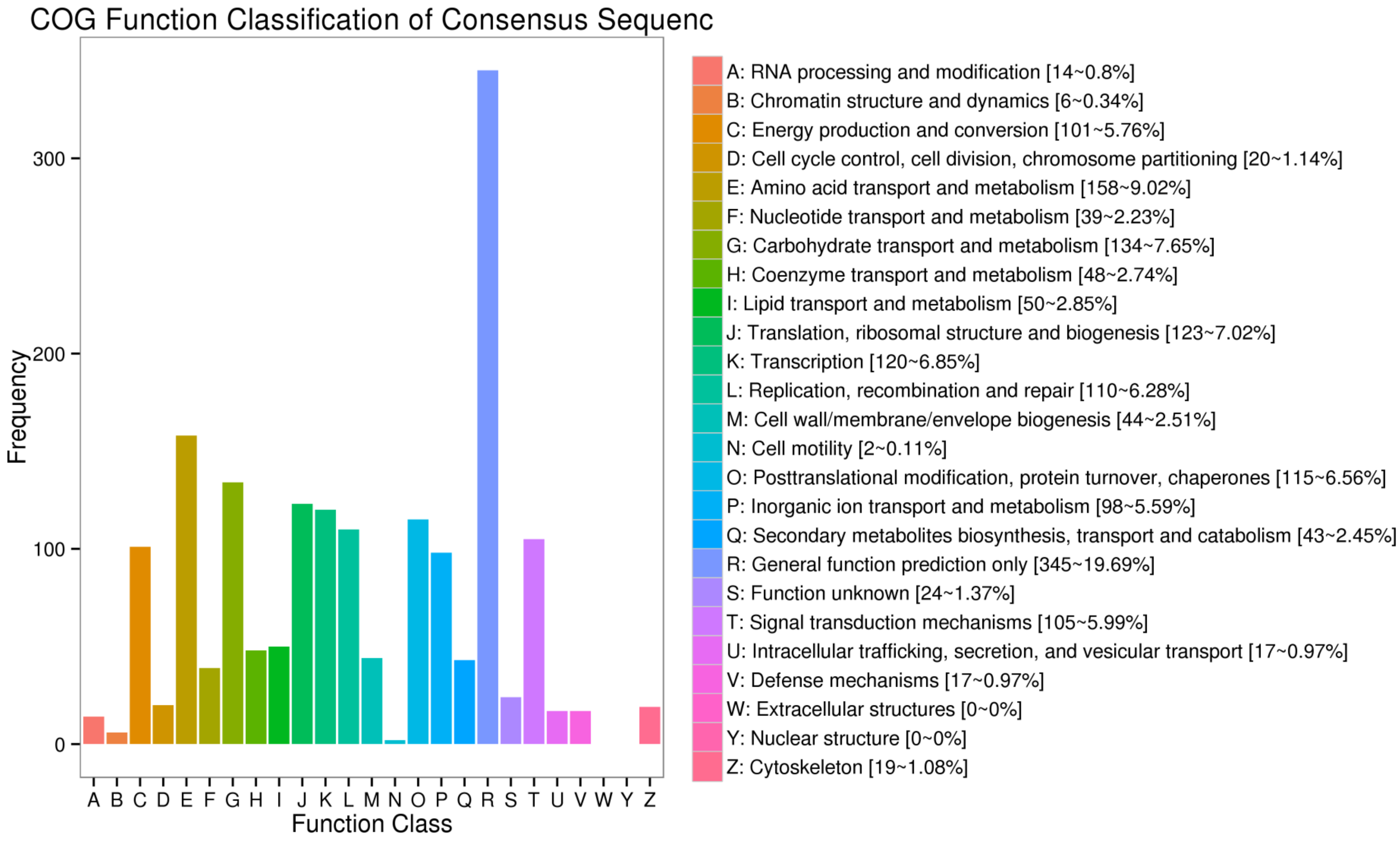

Figure S2: COG functional classification of unigenes

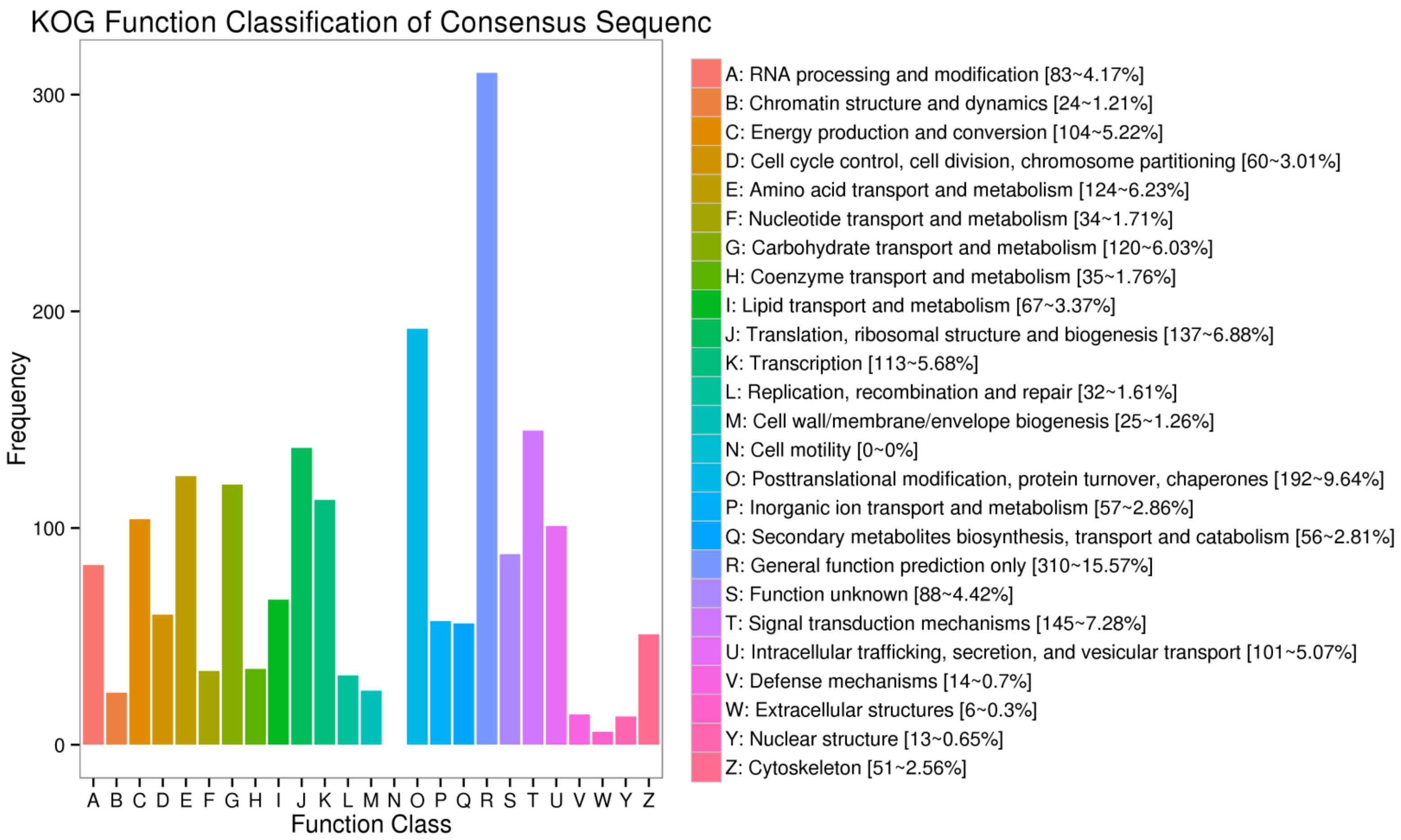

Figure S3: KOG functional classification of unigenes

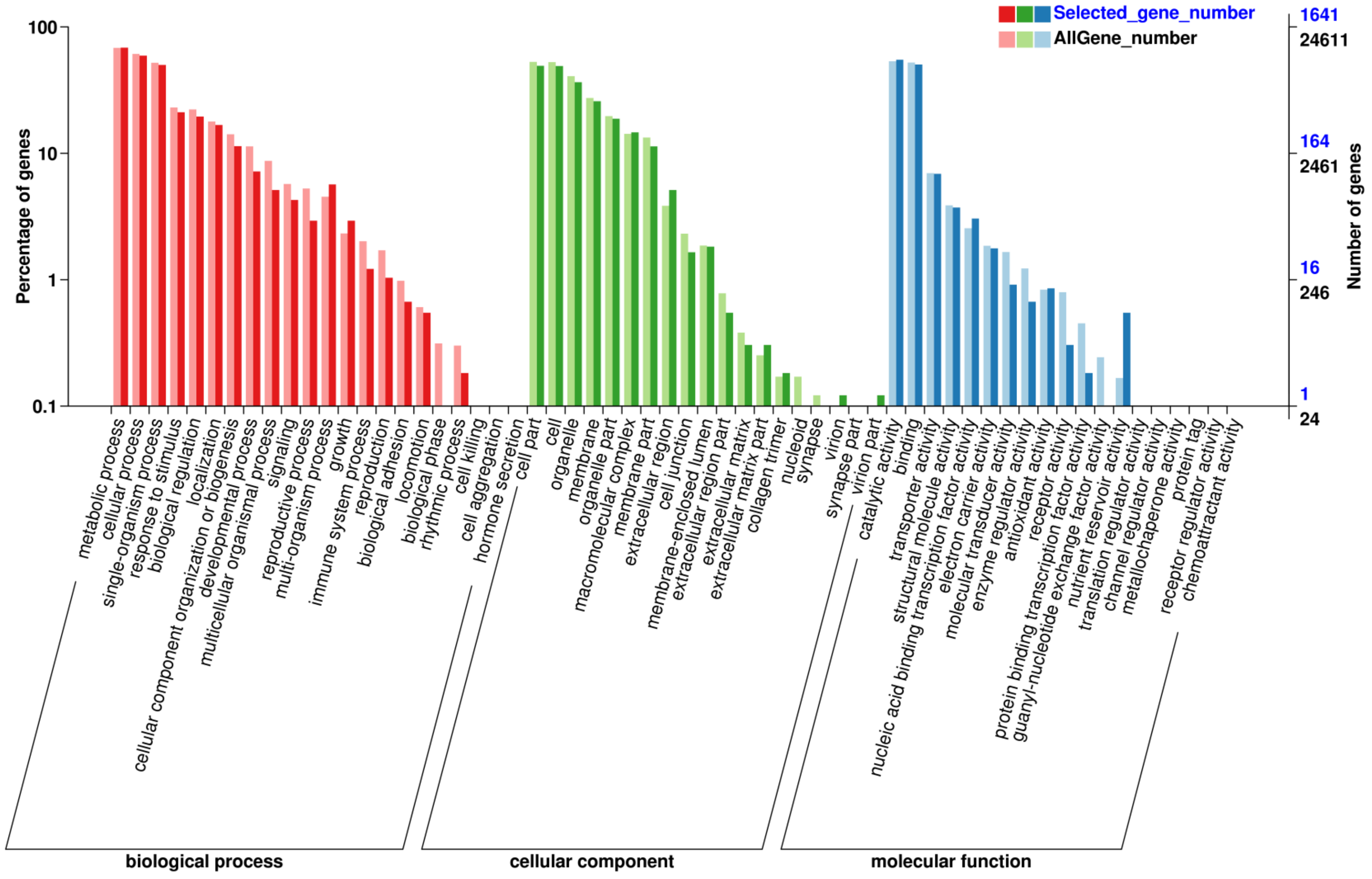

Figure S4: GO functional classification of unigenes
